# Establishment and large-scale validation of a three-dimensional tumor model on an array chip for anticancer drug evaluation

**DOI:** 10.3389/fphar.2022.1032975

**Published:** 2022-10-12

**Authors:** Rong-Rong Xiao, Lei Jin, Nan Xie, Piaopiao Luo, Wenjie Gao, Pengfei Tu, Xiaoni Ai

**Affiliations:** ^1^ R&D Department, Beijing Daxiang Biotech Co., Ltd., Beijing, China; ^2^ Oncology and Immunology Unit, WuXi Biology, WuXi AppTec (Shanghai) Co., Ltd., Shanghai, China; ^3^ State Key Laboratory of Natural and Biomimetic Drugs, School of Pharmaceutical Sciences, Peking University, Beijing, China

**Keywords:** array chip, 3D tumor model, drug evaluation, large-scale validation, *in vivo* predictivity

## Abstract

Two-dimensional (2D) tumor model has always poorly predicted drug response of animal model due to the lack of recapitulation of tumor microenvironment. Establishing a biomimetic, controllable, and cost-effective three-dimensional (3D) model and large-scale validation of its *in vivo* predictivity has shown promise in bridging the gap between the 2D tumor model and animal model. Here, we established a matrigel-based 3D micro-tumor model on an array chip for large-scale anticancer drug evaluation. Compared with the 2D tumor model, the 3D tumor model on the chip showed spheroid morphology, slower proliferation kinetics, and comparable reproducibility. Next, the results of the chemotherapeutic evaluation from 18 drugs against 27 cancer cell lines showed 17.6% of drug resistance on the 3D tumor model. Moreover, the evaluation results of targeted drugs showed expected sensitivity and higher specificity on the 3D tumor model compared with the 2D model. Finally, the evaluation results on the 3D tumor model were more consistent with the *in vivo* cell-derived xenograft model, and excluded 95% false-positive results from the 2D model. Overall, the matrigel-based 3D micro-tumor model on the array chip provides a promising tool to accelerate anticancer drug discovery.

## Introduction

Cancer is one of the fatal diseases worldwide, and pharmaceutical industry is committed to developing novel drugs for cancer therapy. Anticancer drug development is time-consuming and costly that normally takes more than 10 years and 2 billion dollars before it is introduced to the market (Berdigaliyev and Aljofan, 2020). Two-dimensional (2D) *in vitro* tumor model and animal model are widely used in the preclinical drug development. The animal model can predict anticancer drug efficacy by evaluating tumor progression (Ireson et al., 2019). However, it has some limitations of time-consuming and expensive (Pampaloni et al., 2007). More importantly, the species difference makes it difficult to accurately recapitulate human disease phenotypes, and induces clinical failure (Honkala et al., 2022). Compared with the animal model, the *in vitro* 2D model is advantageous for low-cost and high-throughput. However, simplified and homogenized 2D tumor model leads to cell biology and behavior changing dramatically. Cells on the traditional 2D tumor model typically grow on a stiff surface, which often proliferate at a faster rate than *in vivo*, as well as exhibit lamellar, flat and stretched morphologies. Moreover, cells on the 2D model cannot sense the concentration gradient of the medium and extracellular matrix (ECM) components. They are at the same stage of the cell cycle, and show differences in gene/protein expression levels compared with the *in vivo* model. From previous reports, about 30% of the genes expressed differentially on the 2D model compared with the cells *in vivo* (Birgersdotter et al., 2005). The predictivity was compromised by these differences and many drugs showed ineffective when tested in the animal model (Breslin and O'Driscoll, 2013; Carvalho et al., 2017). The 3D tumor model is an emerging *in vitro* model that can better mimic *in vivo* tumor environment. Cells in the 3D model are spherical or aggregated, and proliferate at a slower rate. The 3D architecture generated cells in different growth cycles and exhibited a gene/protein expression profile that is more similar to that of *in vivo* tumor tissue (Edmondson et al., 2014). More importantly, the 3D tumor model better predicted anticancer drug response *in vivo* (Edmondson et al., 2014; Ravi et al., 2015). Though the 3D tumor model has shown promise in bridging the gap between the 2D model and animal model, the superiority of the 3D model is still required further investigation.

The reported 3D tumor models are mainly divided into matrix-based and matrix-free subtypes (Page et al., 2013; Duval et al., 2017). The matrix-free model generates tumor spheroids by hanging drop or low adhesion surface. However, the spheroid formation and procedure of medium exchange are time-consuming. The lack of nutrition and oxygen in the central regions of the spheroid also becomes a concern. Moreover, deeper understanding of tumor microenvironment has identified that the ECM plays an important role in tumor progression and drug response (Weigelt et al., 2010; Habanjar et al., 2021; Paradiso et al., 2021). The ECM not only offers integrins family to anchor cells (Justice et al., 2009; Nirmalanandhan et al., 2010), but also modulates physical properties, such as stiffness, viscoelasticity, and permeability (Eble and Niland, 2019). Previous studies demonstrated that ovarian tumor cells in the ECM-based 3D model were more sensitive to methyltransferase inhibition, however, the 3D cultured osteosarcoma cells showed higher drug resistance and invasiveness behavior (Amatangelo et al., 2013; Monteiro et al., 2020). Matrigel is a widely used mixture of proteins extracted from the mouse sarcomas, containing collagen IV and laminin-111 which provide the scaffold similar to *in vivo* (Driehuis et al., 2020). Nevertheless, the adoption of the matrigel-based 3D tumor model is still hampered because of high consumption of the expensive matrigel, variable matrigel shaping, tedious medium exchange, and lack of large-scale validation for predictivity of *in vivo* response. Previously, we have developed an array chip for prediction of clinical drug-induced liver injury (Xiao et al., 2021). We thus hypothesize this platform is also suitable for generating a biomimetic, reproducible, and cost-effective 3D tumor model to better predict drug response of the animal model.

Herein, we established a 3D tumor model on the array chip, and then validated its predictivity of drug efficacy on a large scale. We firstly compared morphological differences, growth kinetics and result reproducibility between the paired 2D and 3D tumor models. Next, we explored the feasibility of the 3D tumor model for large-scale evaluation of chemotherapeutic and targeted drugs. The evaluation results from the 3D model were systematically compared with the corresponding 2D model. Finally, *in vivo* cell-derived xenograft (CDX) model as a standard was applied to validate the predictive results from the 2D and 3D tumor models. We anticipate this matrigel-based 3D tumor model on the array chip can reduce overuse of the CDX model, as well as accelerate anticancer drug development.

## Materials and methods

### Materials and reagents

Cancer cell lines were purchased from ATCC except for Huh7 (JCRB), PC-9 (Riken BRC), BEL7404 (SUZHOU BEILE BIOTECH), SK-OV-3, A2780, RPMI-8226 (ECACC), HCC78 (DSMZ) and KM-12 (HUATUO). Cell culture medium included RPMI-1640 (#22400-089), McCoy’s 5a (#16600082), DMEM (#11995-065), F-12K (#21127-022) and IMDM (#12440053) was purchased from Gibco. EMEM (#30-2003) and Hybri-Care (#46-X) medium was purchased from ATCC. Leibovitz’s L-15 (#L1518) was purchased from SIGMA. All the medium was contained 10% or 20% FBS (#FND500, ExCell Bio). The 2D and 3D models used the same culture medium. Horse serum (#041241A) was bought from BI. Trypsin (#25200072) and antibiotic-antimycotic (#15240-062) was purchased from Gibco. Dulbecco’s PBS (#21-031-CVC) and matrigel (#354234) were purchased from Corning. DMSO (#D2650) was bought from Sigma. CellTiter-Glo Cell Viability Assay kit (#G7573) and CellTiter-Glo 3D Cell Viability Assay kit (#G9683) were purchased from Promega.

### Design and fabrication of the array chip

The array chip (#IBAC S1, Daxiang Biotech, China) was designed in AutoCAD software. The chip was 127.8 mm long, 85.5 mm wide, and 9 mm center-to-cent spacing according to the geometrical arrangement of the commercial 96-well plate. The top component is the reservoir hole with 6.8 mm diameter and 11 mm height. The middle component is a 3D implanting hole with 2.5 mm diameter and 1.5 mm height. The bottom layer is an ultrathin glass slide. The top and middle components are made of polystyrene and manufactured by injection molding.

### Cell culture

All the cancer cell lines were maintained at 37°C in an atmosphere of 0% or 5% CO2 in flasks and routinely passaged (Supplementary Tables S1, S2). Cells were counted by the hemocytometer with trypan blue staining and harvested in the exponential growth phase for the following experiments.

### Compound stock plate preparation and compound treatment

All the compounds were purchased from SELLECK except for tirapazamine (SIGMA) and T-DM1 (GENENTECH). The compound stock concentrations and working concentrations for dose-response curves were listed in Supplementary Tables S3, S4.

### *In vitro* model establishment and drug administration

For establishment of the 2D model, 135 μL of cell suspension was seeded into the 96-well plate (Greiner, # 655090) and incubated at 37°C, 0% or 5% CO_2_ overnight before drug administration. The optimized cell seeding number for the individual cell line was obtained from the cell growth curves by calculating the doubling time. The doubling time of the same cell line may vary from different labs because of different culture conditions. The next day, we added 15 μL of 10X compound-medium mixture into the 96 well-plate with the final concentration of 0.25% (DMSO) or 1% (saline).

For establishment of the 3D model, the cell resuspension was firstly mixed with the matrigel on ice. Then, 8 μL of the matrigel-cell mixture was added to each well of an array chip (IBAC S1, Daxiang). The array chip was incubated at 37°C for 10 min to solid the matrigel-cell mixture. 150 μL of the culture medium was then added to the reservoir of the chip. The chip was incubated at 37°C, 0% or 5% CO_2_ for 4 days before drug administration. On the 5th day, 115 μL of the culture medium was aspirated. Then, 115 μL of the compound-medium mixture containing 100 μL of fresh medium and 15 μL of 10X compound was added to the reservoir of the chip. The final concentration of the solvent was 0.25% (DMSO) or 1% (saline).

For the primary evaluation, the drugs were administrated by the working concentrations that were decided according to the commonly used dosages in the references (Supplementary Tables S3, S4). For the secondary evaluation, the selected drugs were tested by 9 concentration points at 2 or 5-fold serial dilutions from the working concentrations. The 2D and 3D models were subsequently incubated and treated with the compounds for 3–5 days according to the doubling time. 150 μL of assay medium was used as the blank control for both the 2D and 3D models.

### Cell viability assay and drug screening on the *in vitro* tumor models

Cell viability on the 2D model was detected by the Promega CellTiter-Glo Luminescent Cell Viability Assay Kit (Promega-G7573, whereas those on the 3D model was tested by the Promega CellTiter-Glo 3D Luminescent Cell Viability Assay Kit (Promega-G9683). Briefly, the CellTiter-Glo reagent and the culture medium were mixed with a volume ratio of 1:1. After equilibrating the 96-well plate and array chip at room temperature for approximately 30 min, 75 μL of CellTiter-Glo reagent was added to each reservoir and incubated at room temperature for 45 min to stabilize the luminescent signal. The luminescence intensity (A.U.) was recorded on the 2104 EnVision plate reader. We monitored the luminescence intensity of the cells from Day 1 to Day 7 at different seeding densities, and then the cell growth curves were characterized. The inhibition rate (IR) of the cell viability after drug administration was calculated by the following formula:

IR(%)=(1−(LU compound−LU blank)/(LU control−LU blank))*100%

where LU represents the luminescence intensity detected by the CellTiter-Glo reagent.

Heat map of the inhibition rate of the drugs on the 2D and 3D tumor models from the primary evaluation was plotted. Red color represents higher inhibition rate with stronger efficacy, whereas blue color represents lower inhibition rate with poorer efficacy. Next, the drugs with the inhibition rates between 45% and 95% on both the 2D and 3D tumor models from the primary evaluation were further screened in the secondary evaluation. Dose-response curves and IC_50_ values (μM) were generated by the cell viability against drug concentrations. Heat map of the IC_50_ values of the drugs on the 2D and 3D tumor models from the secondary evaluation was also plotted. Red color represents higher IC_50_ value with poorer potency, whereas blue color represents lower IC_50_ value with stronger potency. Blank boxes in the heat map indicates no test.

### Morphological characterization

After 72 h of culture, the cells were fixed in 4% paraformaldehyde for 20 min, permeabilized in 0.5% Triton X-100 in PBS for 30 min, and then blocked in 5% bovine serum albumin (BSA) in PBST (0.1% Tween 20) for 30 min. Then the cells were incubated with 5 μg/ml fluorescein isothiocyanate (FITC)-phalloidin for 30 min at room temperature, whereas the nuclei were stained with DAPI. Phase-contrast and fluorescence images were captured using an inverted fluorescence microscope (Olympus IX73, Tokyo, Japan) and a confocal laser-scanning microscope (Eclipse Ti2, Nikon).

Live/dead assay was performed using a Live/dead cell Viability Assay Kit (Invitrogen). Nonfluorescent calcein-AM is hydrolysed by the live cells into green fluorescent calcein, whereas ethidium homodimer-1 can only pass through the membrane of the dead cells. The cells were rinsed twice with PBS before the fluorochromes were added and incubated for 45 min. Fluorescence images were captured using an inverted fluorescence microscope (Olympus IX73, Tokyo, Japan).

### Drug efficacy on the cell-derived xenograft model

The formulation and any modification of this protocol had been approved by the Institutional Animal Care and Use Committee (IACUC) of WuXi AppTec (Shanghai, China). The use and welfare of laboratory animals were governed by the Regulations of the Association for Assessment and Accreditation of Laboratory Animal Care (AAALAC). Routine monitoring of the animal’s health and mortality was performed, including the effects of tumor growth and medication on the animal’s daily behavior such as activity, food and water intake (visual only), changes in body weight (weight measured twice a week), physical signs, or other abnormalities. Animal deaths and side effects were recorded based on the number of animals in each group.

Human tumor cells were cultured *in vitro* at 37°C under 5% CO_2_ conditions. The culture medium was supplemented by 10% fetal bovine serum (FBS), 100 U/mL penicillin and 100 μg/ml streptomycin. Cells were passaged two to three times per week. When the cell confluence reached 80%–90%, the cells were collected, counted and inoculated. Tumor cells were subcutaneously inoculated into the right-back of each BALB/c nude mouse, and the drug administration began when the average tumor volume reached about 150 ± 50 mm^3^. Animals were divided randomly consisting of six mice per group (day 0). Drug were administrated from day 0 according to the literatures and recommendation from WuXi AppTec (Supplementary Table S5). In this study, we evaluated the correlation of the drug response between the *in vitro* cell models and *in vivo* CDX model with different compound and cell line combinations.

### Tumor measurement *in vivo*

Tumor diameter was measured twice a week with a vernier calliper. The tumor volume was calculated as:

V=0.5 a×b2

where a and b represent the long-axis diameter and short-axis diameter of the tumor, respectively.

The tumor growth curves on the *in vivo* CDX model with or without drug treatment were recorded according to the tumor volume with time. The tumor volume at day n was normalized by the tumor volume at day 0. Each group had six mice. Cmax is the maximal plasma concentration after the drug administration. The evaluation of the drug efficacy on the *in vivo* CDX model was determined by T/C (%) or TGI (%) that calculated by the following formula:

T/C(%)=Mean tumor volume measured in the drug treatment group/Mean tumor volume measured in the control group×100

TGI(%)=[1−Mean tumor volume measured in the drug treatment group/Mean tumor volume measured in the control group]×100

### Data analysis

During the chemotherapeutic and targeted drug screening, the percent coefficient of variation (CV) and Z′ factor were calculated by the following formula:

CV=ó/X

Where ó = Standard deviation, and X = Mean of replicate

Z′factor=1−(3*(ó n+ó p)/(X n−X p)

Where n = negative control, and p = positive control

The drug with the highest efficacy was used as positive control for each plate or chip, whereas vehicle control was used as negative control. All the array chips and 96-well plates were used to calculate the values of CV and Z′ factor by detection of the cell viability.

The data were imported into GraphPad Prism 9.0 software (San Diego, CA) to conduct statistical analysis. Values are presented as means ± standard deviation (S.D.) of three replicates. Statistical analysis was performed using a two-tailed Student’s t test. The asterisks * and *** denote statistical significance with *p* values of less than 0.05 and 0.001, respectively.

## Results

### Construction of tumor models for anticancer drug evaluation

**FIGURE 1:**
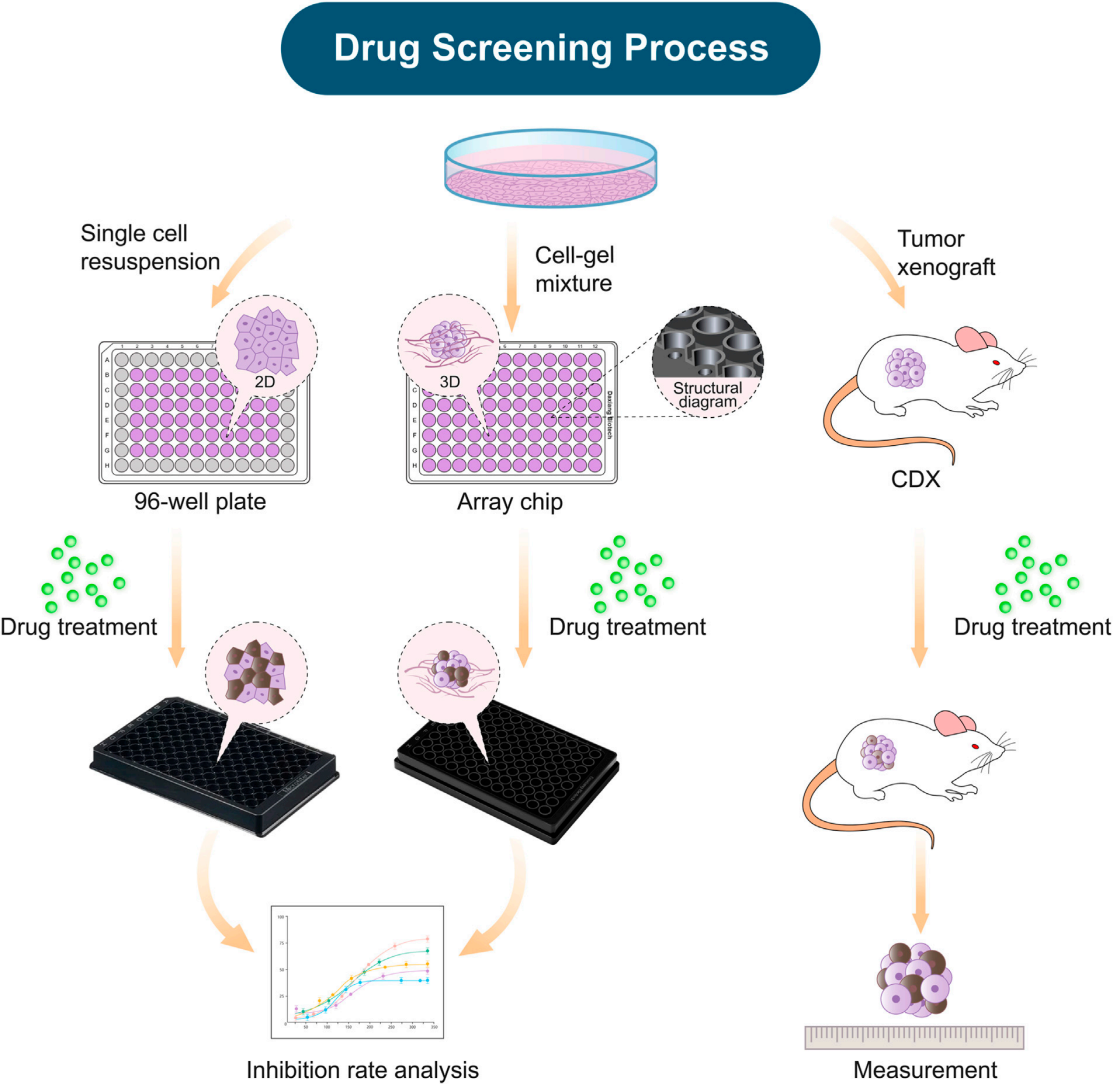
Schematic diagram demonstrating the construction of *in vitro* and xenograft tumor models for drug efficacy evaluation. The establishment of the tumor models, including the 2D model on the 96-well plate, the 3D model on the array chip, and the cell-derived xenograft model (CDX). All these tumor models were applied for evaluation of anticancer drug efficacy.

In this study, we established three kinds of tumor models for anticancer drug evaluation, including the 2D tumor model on the conventional 96-well plate, the 3D tumor model on the array chip (Xiao et al., 2021), and cell-derived xenograft (CDX) model (Figure 1). The 3D tumor model was generated by inoculating 8 μL matrigel-cell mixture into the 3D implanting hole of the array chip. The 3D implanting hole on the chip provides the uniform and controlled shaping of the matrigel that allows the 3D model to keep the fixed location and shape. The nested design of the chip reduces the cells and matrigel consumption, as well as provides convenience for medium exchange without disturbing the matrigel shaping. The geometrical arrangement of the chip is designed as a standard plate and compatible with various high-throughput devices. The ultra-thin optical transparent glass underneath and black plastic material are specially designed for high-quality fluorescence imaging. There is an anti-evaporation chamber around the reservoir holes on the chip. PBS can be added to the chamber to minimize the evaporation during culturing, which contributes to the usage of all the 96 units. Drugs were diluted to the desired concentrations, and then added to the 2D or 3D tumor model at the desired time. After incubation of 3–5 days, cell viability was detected by CellTiter-Glo reagent under a micro-plate reader. In this study, CDX model as a standard was applied to further validate the predictive results from the 2D and 3D *in vitro* tumor models.

### Morphological comparison of tumor cells between the paired 2D and 3D models

**FIGURE 2:**
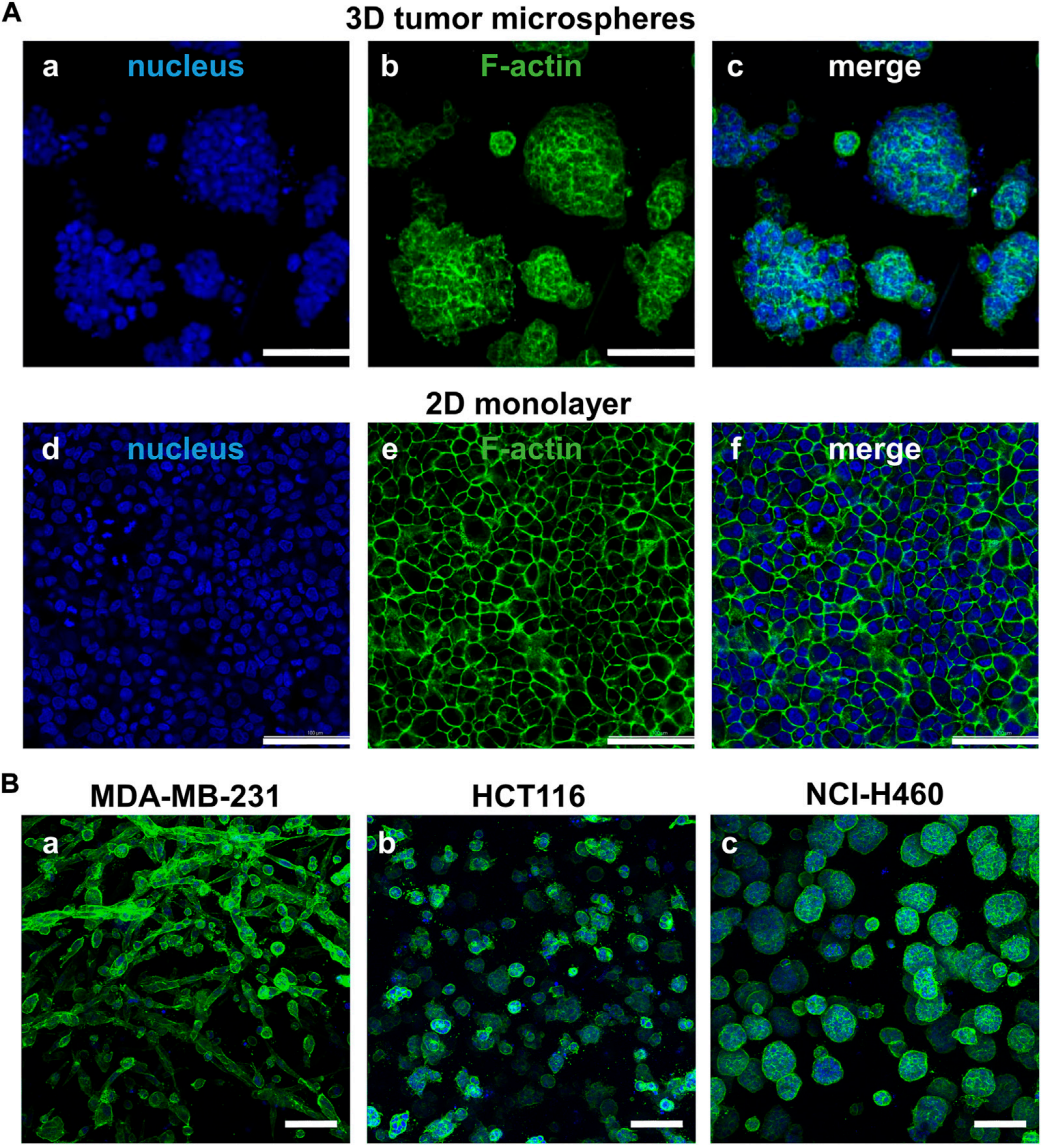
Comparison of cell morphology between the paired 2D models and 3D models. **(A)** Different cell morphology of NCI-H460 cells cultured on the 2D and 3D models staining by F-actin (green) and nuclei (blue). **(B)** The cell morphology of three representative cell lines cultured on the 3D tumor model. Scale bar = 100 µm.

As shown in Figure 2, we firstly compared morphology differences of the tumor cells cultured on the 2D and 3D models. After 72 h of incubation, the cell morphology was observed by a confocal microscope with phalloidin-labeled F-actin and DAPI-labeled nucleus. The cells on the 2D model were well-spread and flatten, whereas the cells on the 3D model were compactly aggregated. The fluorescent staining of MDA-MB-231, HCT116, and NC-H460 cells cultured on the 3D model revealed different morphologies. The MDA-MB-231 cells grew in strips, whereas the HCT116 and NC-H460 cells formed tight spheroids with different diameters.

### Comparison of growth kinetics and reproducibility between the paired 2D and 3D models

**FIGURE 3:**
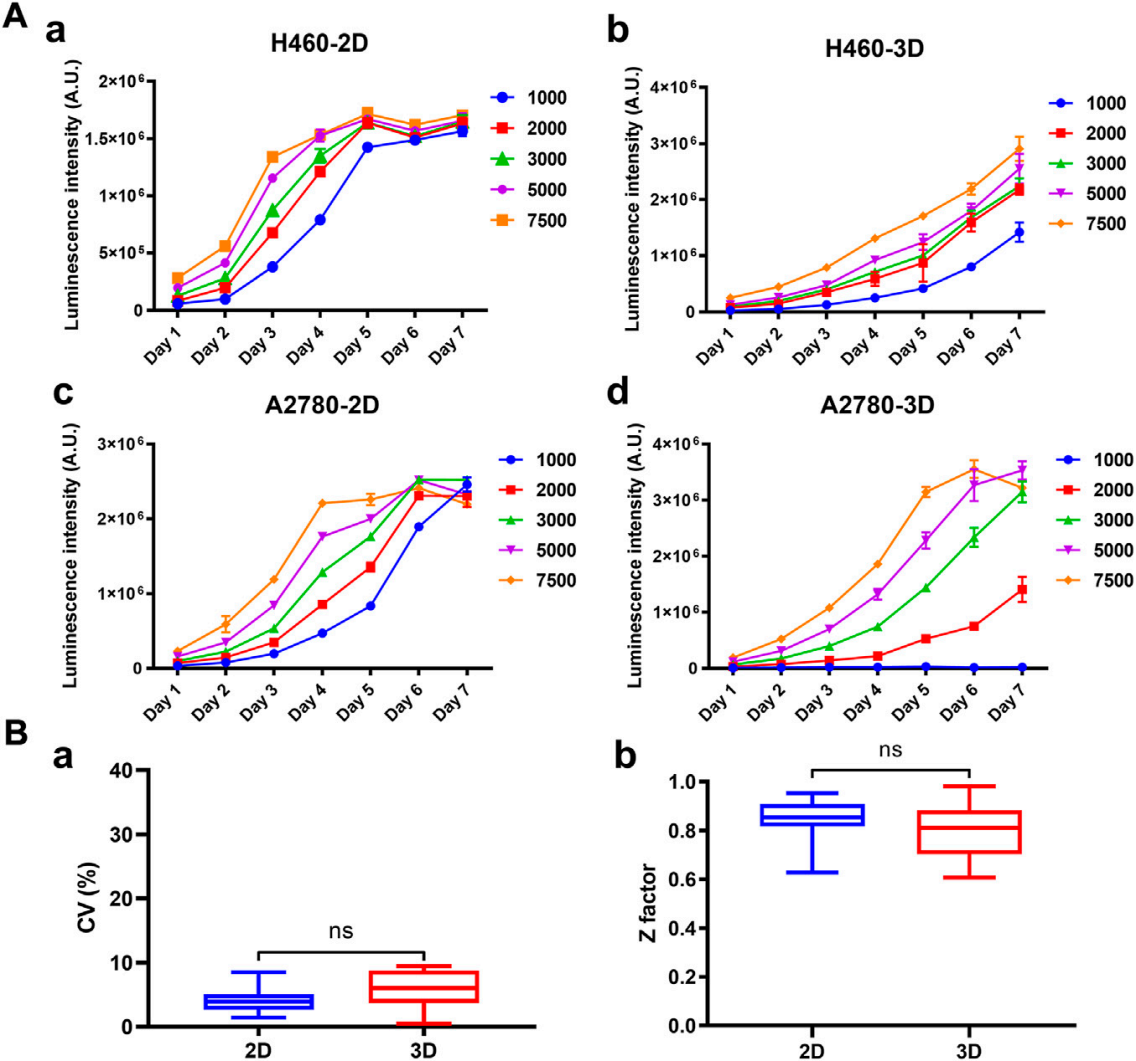
Growth curves and robustness of the 2D and 3D models. **(A)** Cell growth curves of H460 and A2780 cells cultured on the 2D and 3D models were characterized at different seeding densities. Cell viability was indicated by luminescence intensity measured using CellTiter-Glo. **(B)** Comparison of the statistics values of coefficient of variation (CV) and Z′ factor between the 2D and 3D models. All data are presented as means ± SD of three replicates.

The growth curves of 39 cancer cell lines cultured on the array chip were compared with their 2D counterparts. Cell viability was determined by ATP quantification detected by CellTiter-Glo reagent. As shown in Figure 3A and Supplementary Figure S1, the luminescence intensity was steadily increased from day 1 to day 7 on the 3D tumor model. However, the cells usually grew rapidly at the beginning and then entered a plateau phase on the 2D model. The doubling time (DT) was calculated according to the growth curves. By comparing the doubling time of the 2D and 3D models with the same cell seeding number, 38.4% (15 out of 39) of the cell lines grew slower on the 3D model with a DT ratio higher than 1.1 (Supplementary Figure S2), whereas 35.9% (14 out of 39) of the cell lines had similar growth rate with a DT ratio between 0.9 and 1.1. Herein, we used the matrigel as the basement membrane matrix to construct the 3D tumor model. The growth curves of the tumor cells may vary depending on the different protein components of the hydrogels. Next, the effect of the pre-culture time on the drug response was investigated (Supplementary Figure S3). Three representative drugs, including doxorubicin (DOX), epirubicin and vinorelbine, were exerted on the 3D cultured NCI-H460 cells after pre-culturing for 24 h or 96 h. There was a >3-fold shift in the IC_50_ values for the vinorelbine between the 24 h and 96 h of the pre-culture. However, there was no significant shift of the IC_50_ value for the DOX and epirubicin. The IC50 values for the DOX, epirubicin, and vinorelbine after 96 h preincubation were 0.10 μM, 0.12 μM, and 0.20 μM, respectively. The results indicated that the drug potency was affected by the pre-culture time when it was not too effective. The formation of microspheres was also considered to be more comparable to the *in vivo* situation. We therefore chose 96 h pre-culture time to better recapitulate drug resistance *in vivo*. To further investigate the reproducibility of the 3D tumor model, the coefficient of variation (CV) of the cell viability and Z′ factor was calculated from all the array chips or 96-well plates. The statistic value of the average CV was below 10% and the Z′ factor was above 0.7 on the 3D model, which were comparable to the corresponding 2D model (Figure 3B). Therefore, this matrigel-based 3D tumor model on the array chip met the requirements of high-throughput drug evaluation.

### Comparison of chemotherapeutic evaluation between the paired 2D and 3D models

**FIGURE 4:**
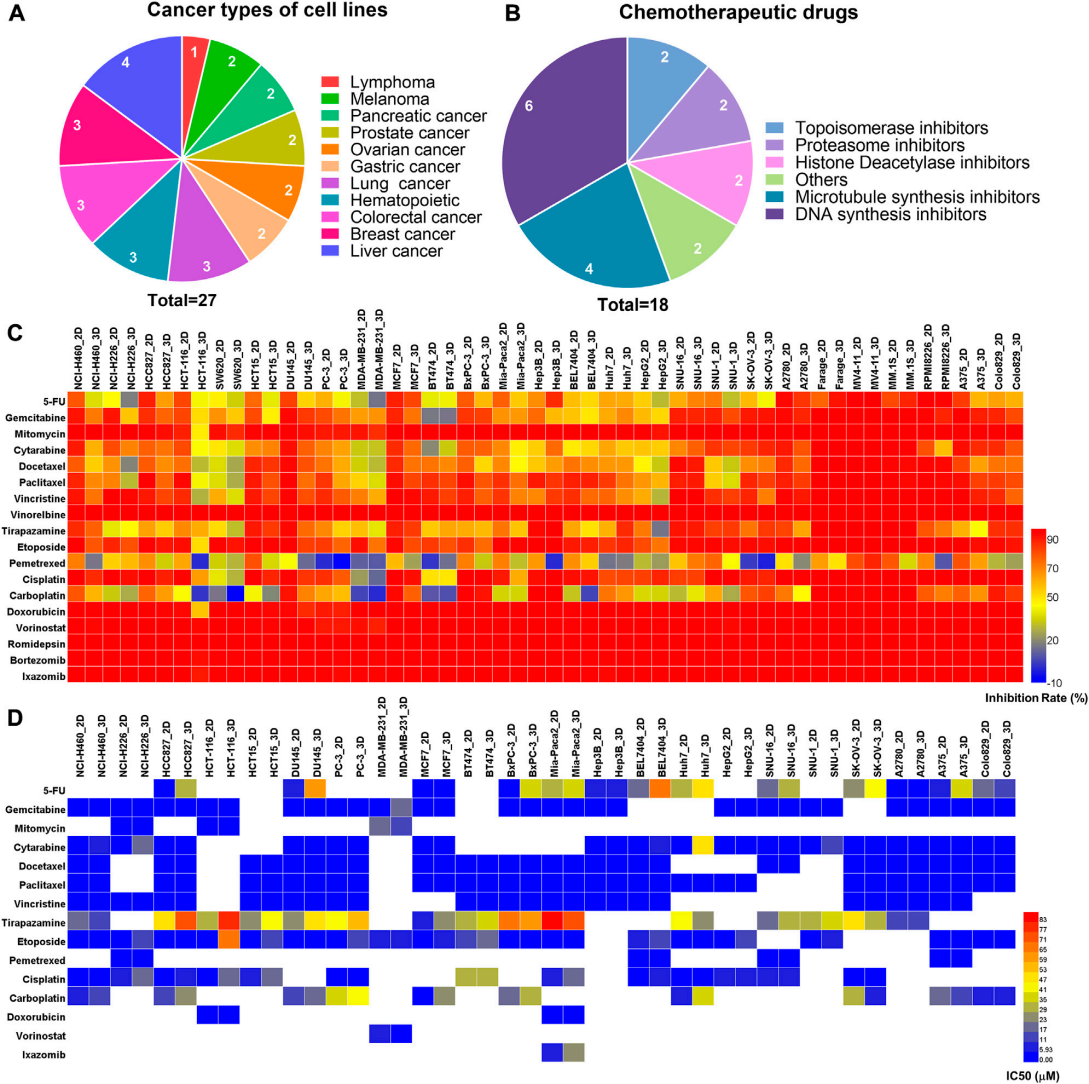
The comparison of primary and secondary evaluation of chemotherapy drugs on the paired 2D and 3D models. **(A)** Schematics of distribution of the human cancer cell lines with different cancer types in this study. **(B)** Schematics of distribution of the chemotherapy drugs with different mechanisms of action in this study. **(C)** Heat map of the inhibition rates of the drugs on the 2D and 3D tumor models from the primary evaluation. Red color represents higher inhibition rate with stronger efficacy, whereas blue color represents lower inhibition rate with poorer efficacy. **(D)** Heat map of the IC_50_ values of the drugs on the 2D and 3D tumor models from the secondary evaluation. The IC_50_ values were calculated from the dose-response curves. Red color represents higher IC_50_ value with poorer potency, whereas blue color represents lower IC_50_ value with stronger potency. Blank boxes in the heat map indicates no test.

We firstly assessed the performance of the 3D tumor model for chemotherapeutic evaluation. We chose a panel of 27 human cancer cell lines from 11 organ origins, including gastric cancer, lymphoma, pancreatic cancer, ovarian cancer, prostate cancer, melanoma, hematopoietic, liver cancer, lung cancer, colorectal cancer, breast cancer (Figure 4A). The detailed information of the human cell lines was shown in Supplementary Table S1. 18 FDA-approved chemotherapy drugs were selected for high-throughput evaluation (Figure 4B, Supplementary Table S2). The anticancer efficacy of the drugs was systematically compared between the 2D model and 3D model.

On the primary evaluation, a total of 486 groups of data were obtained after being treated with the highest working concentration of the drugs. There were some differences in the drug response between the 2D and 3D models (Figure 4C). Setting 50% cell inhibition rate (IR) as a cutoff, 8.6% (42/486) of the drugs showed ineffective on the 3D tumor model, whereas those exhibited effective on the corresponding 2D tumor model. Among the effective drugs on two models, 27.4% (113/411) of the drugs showed more resistance on the 3D tumor model with the IR difference more than 5% (Supplementary Figure S4). The representative results from the primary evaluation exhibited the drug resistance on the 3D tumor model (Supplementary Figure S5). The drug efficacy could also be high-throughput evaluated *in situ* by immunofluorescence detection on the array chip. We performed a live/dead assay on the 3D cultured HCT116 and H460 cells after being treated by the DOX. The live cells were stained in green, and the dead cells were in red (Supplementary Figure S6). The percentage of the dead cells in the drug treatment groups were significantly higher than that in negative control groups.

Next, the drugs with the inhibition rates between 45% and 95% on the primary evaluation were further determined their IC_50_ values in the secondary evaluation. The IC_50_ heat map from the 160 groups of data further showed the drug resistance on the 3D tumor model (Figure 4D). The IC_50_ ratio was calculated by the IC_50_ value on the 3D model divided by the value on the 2D model. 27.5% (44/160) of the drugs showed high resistance on the 3D model with the IC_50_ ratio >5, and 28.7% (46/160) of the drugs showed low resistance with the IC_50_ ratio of more than 2 but less than 5 (Supplementary Figure S7). The higher IC50 ratio indicated less sensitivity on the 3D model. As an example, the IC_50_ values of the cytarabine on the 3D cultured NCI-H460 and A2780 cells were 44-fold and 114-fold higher than the corresponding 2D models, which were 0.14/6.18 μM (2D/3D) and 0.04/4.57 μM (2D/3D), respectively (Supplementary Figure S8).

In summary, 17.6% (86/486) of the chemotherapeutic drugs showed higher resistance on the 3D tumor model, including 42 groups of data from the primary evaluation and 44 groups of data from the secondary evaluation. The drug resistance on the 3D tumor model indicated that the 2D tumor model may generate false-positive results.

### Comparison of evaluation of targeted drugs between the paired 2D and 3D models

**FIGURE 5:**
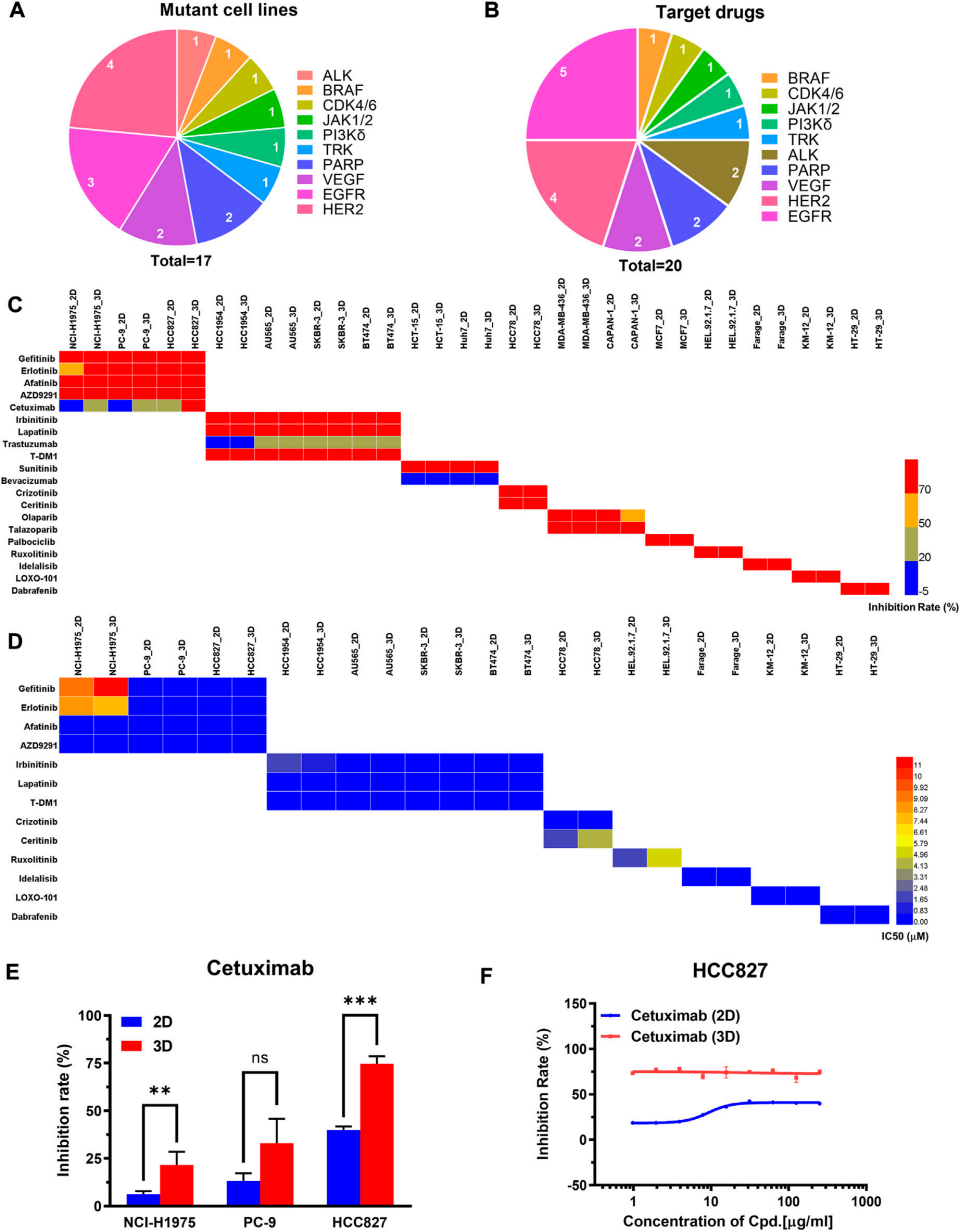
The comparison of primary and secondary evaluation of targeted drugs between the paired 2D and 3D models. **(A)** Schematics of distribution of the human cancer cell lines with different mutant genes in this study. **(B)** Schematics of distribution of targeted drugs with different mutant genes in this study. **(C)** Heat map of inhibition rates of the drugs on the 2D and 3D models from the primary evaluation. Red color represents higher inhibition rate with stronger efficacy, whereas blue color represents lower inhibition rate with poorer efficacy. Blank boxes in the heat map indicates no test. **(D)** Heat map of the IC_50_ values of the drugs on the 2D and 3D models from the secondary evaluation. The IC_50_ values were calculated from the dose-response curves. Red color represents higher IC_50_ value with poorer potency, whereas blue color represents lower IC_50_ value with stronger potency. Blank boxes in the heat map indicates no test. **(E)** Comparison of inhibition rates of the EGFR targeting cetuximab on the EGFR-mutated cell lines (NCI-H1975, PC-9, HCC827) cultured on 2D and 3D models. The working concentration of the cetuximab was 250 μg/ml. **p < 0.01, ***p < 0.001, non-significant (ns) *p* > 0.05. **(F)** Representative dose-response curves of cetuximab on the 2D and 3D models of HCC827. The cetuximab was tested by 9 concentration points at 2-fold serial dilution, including 250, 125, 62.5, 31.25, 15.62, 7.81, 3.91, 1.95, 0.97 μg/ml. All data are presented as means ± SD of three replicates.

We next assessed the performance of the 3D tumor model for screening of the targeted drugs. We selected 17 human cancer cell lines with known mutant genes, such as EGFR, VEGF, BRAF, ALK, and HER2 (Figure 5A). 20 clinical drugs targeting the corresponding mutant genes were administrated on the cells (Figure 5B). On the primary evaluation, we explored the inhibition rates of the targeted drugs with the highest working concentrations on the 2D and 3D models (Supplementary Table S4). On the two models, the inhibition rates were similar except for 10 groups of data exhibited >5% differences (Figure 5C). On the secondary evaluation, all the IC50 ratios from the parental 2D and 3D models were less than 5-fold differences except for two cases (Figure 5D). We found that the EGFR-targeting cetuximab was more sensitive on the 3D model to all the EGFR mutant lung cell lines, including HCC827, NCI-H1975, and PC-9 (Figure 5E, Supplementary Figure S9, Supplementary Table S6). The IC50 value on the 3D-HCC827 model was <0.97 μM, whereas that on the corresponding 2D model was >250 μM (Figure 5F). Interestingly, other EGFR-targeting drugs, including gefitinib, erlotinib, afatinib, AZD9291, also showed similar results except for the gefitinib on the NCI-H1975 cells (Supplementary Table S6). The increased sensitivity of the cells cultured on the 3D model to the drugs targeting EGFR suggested that the EGFR expression and downstream signaling may be altered under different culture conditions (Howes et al., 2014; Ayuso et al., 2019). The other case was the HER2-targeting T-DM1 on the HCC1954 cells (HER2-positive). The IC50 value of the T-DM1 on the 3D model showed 8-fold higher than the corresponding 2D model, which were 0.16 μM and 0.02 μM, respectively. The T-DM1 is an antibody-drug conjugate (ADC) that delivers the DM1 chemotherapy specifically to the HER2-positive breast cancer cell (Barok et al., 2014). The increased resistance of the T-DM1 on the 3D tumor model probably due to its chemotherapeutic mechanism of action.

The specificity also contributes to the drug’s therapeutic potency. The targeted drugs were expected to act on the specific targets without unwanted toxicities and off-target effects (Zhao et al., 2020). Herein, we compared the specificity of the drug response between the 2D model and the 3D model. 6 targets were selected to implement the cross-evaluation comparison, including EGFR, HER2, ALK, PARP, CDK4/6, and BRAF. We found HER2 and EGFR targets showed improved specificity on the 3D model. Taking the EGFR expressed model as an example, the 3D tumor model showed a slightly higher sensitivity to the EGFR targeted drug of the gefitinib. The 3D model exhibited much stronger resistance to the non-EGFR targeted drug of the talazoparib with a 9-fold increase of the IC50 value than the corresponding 2D model. Compared with the 2D model, the EGFR expressed 3D model improved the 26-fold specificity (Supplementary Figure S10). Moreover, the HER2 expressed model exhibited similar results. The 2D and 3D models showed similar sensitivity to the HER2 targeted drug of the irbinitinib. The 3D model showed higher resistance to the non-HER2 targeted drugs of the olaparib and talazoparib with 2.5-fold and 11-fold increases of the IC_50_ values than the corresponding 2D model (Supplementary Figure S11). These results indicated that the 3D model may reduce off-target effects.

Taken together, the evaluation results of targeted drugs showed expected sensitivity and higher specificity on the 3D tumor model, indicating the 2D model may generate the false-positive results.

### The 3D tumor model on the array chip was more consistent with *in vivo* CDX model for anticancer drug evaluation

**FIGURE 6:**
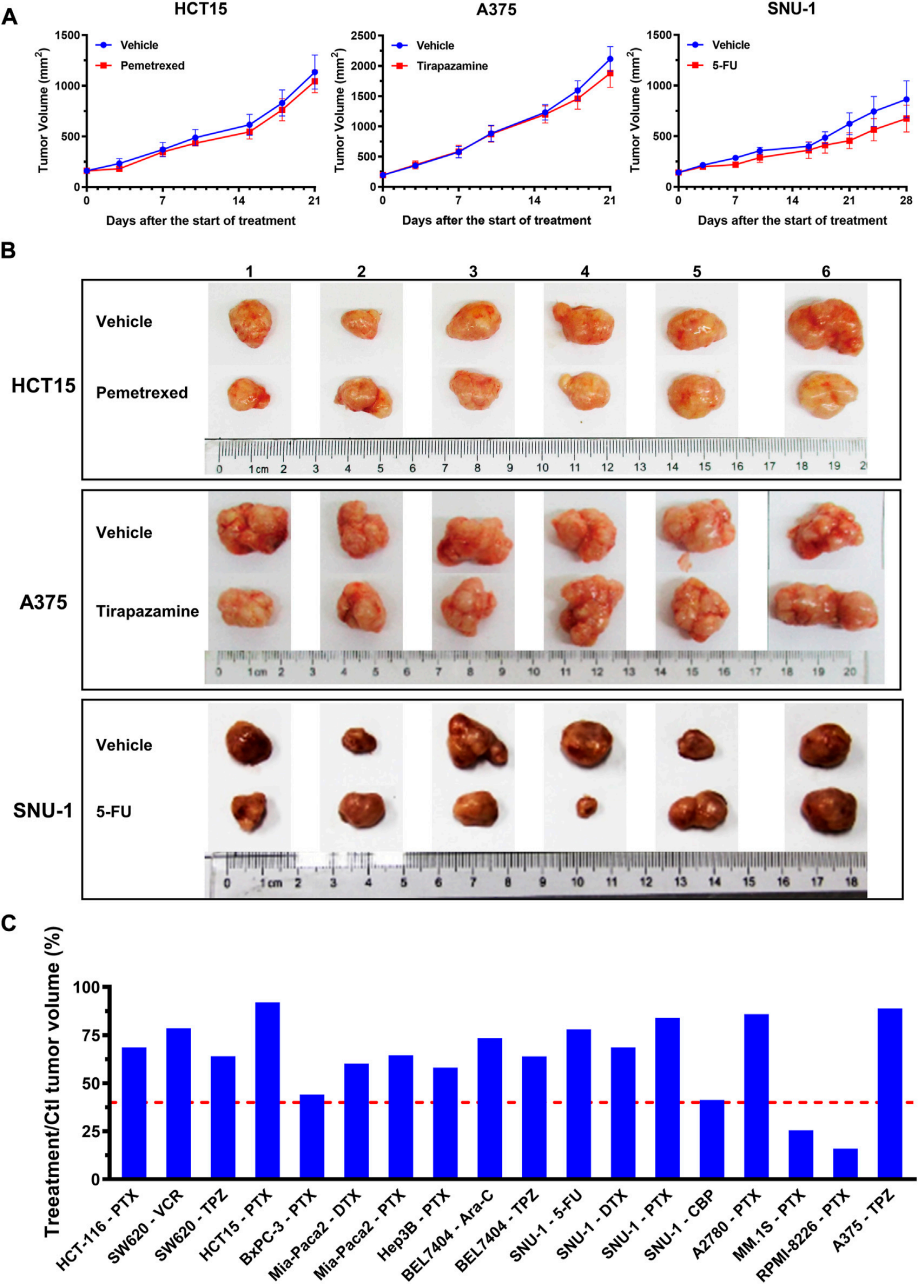
Drug efficacy evaluation on the *in vivo* CDX model. **(A)** Tumor growth curves on the *in vivo* CDX model with or without drug treatment. Pemetrexed, tirapazamine and 5-FU were injected into the HCT15, A375 and SNU-1 xenografted models, respectively. Values are presented as means ± S.D. **(B)** Images of excised tumor tissues with or without drug treatment after sacrificing the mice at the end of the experiments. **(C)** The *in vivo* T/C values from our experiments. The T/C threshold of 40% was labeled in the red dotted line.

To further verify the 3D tumor model to resemble the *in vivo* experimental outcomes, we investigated the representative drug efficacy on the cell-derived xenograft (CDX) model. 40 groups of data were selected from the evaluation results that showed effective on the 2D model, however, exhibited ineffective on the corresponding 3D tumor model. T/C > 40% or TGI <60% on the CDX model was set as a threshold to determine the drug effectiveness as previous reports (Merriman et al., 1996; Teicher, 2013). In 38 groups, the suppression of the tumor growth after the drug treatment was not significant compared with that in the control group. For example, on the HCT15 xenografted model, the tumor volume in the control group was 1135 ± 168 mm^3^, whereas that on the pemetrexed-treated group was 1044 ± 112 mm3 after 21 days. Tumor regression (T/C) on the pemetrexed-treated HCT15 xenografted model was calculated to be 92%. The higher T/C values were also observed on the tirapazamine-treated A375 model with 88.9%, and the fluorouracil (5-FU)-treated SNU-1 model with 78% (Figures 6A,B). The *in vivo* T/C values from our study and previous reports were summarized in Table 1. According to the T/C and TGI thresholds, only 2 out of 40 tests showed effective on the CDX model, which are the pemetrexed-treated MM.1S xenografted model and PRMI-8226 xenografted model (Figure 6C). The efficacy results from the 2D model, 3D model, and CDX model are also summarized in Table 1. The drug responses on the 3D tumor model were more consistent with the *in vivo* CDX model, and 95% (38 out of 40) of the false-positive results on the 2D tumor model was excluded. Therefore, our 3D tumor model on the array chip could better predict *in vivo* drug response than the corresponding 2D tumor model.

**TABLE 1:** *In vivo* T/C or TGI values and the evaluation results from three models.

	Cell line	Drugs	T/C (%)	TGI (%)	Effective		
					CDX model	2D model	3D model
1	NCI-H460	5-FU		24.6	N Bi et al. (2014)	Y	N
2	NCI-H460	Pemetrexed		∼0	N Izbicka et al. (2009)	Y	N
3	HCC827	Pemetrexed		41.5	N Cui et al. (2018)	Y	N
4	HCT-116	5-FU	>40		N Guo et al. (2006)	Y	N
5	HCT-116	Cytarabine	>40		N Waud et al. (2003)	Y	N
6	HCT-116	Docetaxel	>40		N Guo et al. (2006)	Y	N
7	HCT-116	Paclitaxel	>40		N Kim et al. (2014)	Y	N
8	HCT-116	Vincristine	57		N Yamori et al. (1999)	Y	N
9	HCT-116	Pemetrexed	68.6		N	Y	N
10	SW620	Cytarabine	>40		N Waud et al. (2003)	Y	N
11	SW620	Vincristine	78.5		N	Y	N
12	SW620	Tirapazamine	64		N	Y	N
13	HCT15	5-FU	61		N Yamori et al. (1999)	Y	N
14	HCT15	Gemcitabine		25.3	N Qin et al. (2014)	Y	N
15	HCT15	Pemetrexed	92		N	Y	N
16	PC-3	5-FU		20	N Yee et al. (1998)	Y	N
17	MDA-MB-231	Paclitaxel	>40		N Samanta et al. (2014)	Y	N
18	MDA-MB-231	Tirapazamine	>40		N Emmenegger et al. (2006)	Y	N
19	MCF7	Pemetrexed	76.4		N Bai et al. (2018)	Y	N
20	BxPC-3	Pemetrexed	44.1		N	Y	N
21	Mia-Paca2	Cytarabine		7	N Merriman et al. (1996)	Y	N
22	Mia-Paca2	Docetaxel	60.2		N	Y	N
23	Mia-Paca2	Pemetrexed	64.5		N	Y	N
24	Hep3B	Pemetrexed	58.1		N	Y	N
25	BEL7404	Cytarabine	73.4		N	Y	N
26	BEL7404	Tirapazamine	63.9		N	Y	N
27	Hep G2	5-FU	>40		N Wang et al. (2016)	Y	N
28	Hep G2	Vincristine	74.3		N Sun et al. (2009)	Y	N
29	Hep G2	Tirapazamine	51.5		N Zhang et al. (2010)	Y	N
30	SNU-1	Paclitaxel		43.1	N Jung et al. (2019)	Y	N
31	SNU-1	5-FU	78.0		N	Y	N
32	SNU-1	Docetaxel	68.6		N	Y	N
33	SNU-1	Pemetrexed	84.0		N	Y	N
34	SNU-1	Carboplatin	41.2		N	Y	N
35	SK-OV-3	5-FU	75		N Yamori et al. (1999)	Y	N
36	A2780	Carboplatin	67		N Banerji et al. (2008)	Y	N
37	A2780	Pemetrexed	85.9		N	Y	N
38	MM.1S	Pemetrexed	25.5		Y	Y	N
39	RPMI-8226	Pemetrexed	15.9		Y	Y	N
40	A375	Tirapazamine	88.9		N	Y	N

Note: Drug efficacy *in vivo* indicated by T/C or TGI was from our experiment or the literature. Y: effective; N: ineffective.

## Discussion

During the anticancer drug development, the widely used 2D model has compromised accuracy to predict *in vivo* drug response due to different cell biology (Birgersdotter et al., 2005; Breslin and O'Driscoll, 2013; Carvalho et al., 2017). In this study, we established a bio-mimetic, controllable, and cost-effective matrigel-based 3D tumor model on an array chip. The 3D tumor model had more physiological relevance and improved *in vivo* predictivity of drug efficacy than the 2D model. Compared with the 2D model, the 3D model showed different cell morphology and slower proliferation kinetics, which were more similar to *in vivo* tumor.

The 3D tumor model was established on the array chip. Compared with the traditional matrigel assay, the array chip provides microscaled and controlled shaping of the matrigel that allows the 3D tumor model to reduce the cost by more than 50% and achieves high reproducibility (CV < 10%). The traditional matrigel assay usually reduced matrigel concentration (5% vol/vol) for drug testing to improve reproducibility (Driehuis et al., 2020). The different concentrations of the matrigel may alter the composition of secretion proteins, cellular phenotype, and drug response (Cui et al., 2022). The high reproducibility of the 3D tumor model using the high concentration of matrigel (>65% vol/vol) is because of controlled shaping of the matrigel and medium exchange without disturbing the matrigel. The good value of Z′ factor (>0.7) indicated that this 3D model is suitable for high-throughput drug screening.

Moreover, the feasibility of the 3D tumor model on the array chip for predicting drug efficacy on the *in vivo* CDX model was verified on a large scale. Previous papers have compared 2D and 3D models in drug efficacy prediction on small pilot studies (Nirmalanandhan et al., 2010; Weigelt et al., 2010; Longati et al., 2013; Imamura et al., 2015). In our study, the results showed that the cell culture conditions had a significant impact on the drug response, and the influence on the chemotherapeutic and targeted drugs was different based on a large panel of drugs and cancer cell lines. The evaluation results from the chemotherapeutic drugs showed higher resistance on the 3D tumor model. It is note that the different drug response between the 2D and 3D model was related to the choice of the drugs and their anticancer efficacy. In this study, we chose the chemotherapeutic drugs that are commonly used in the clinical practice to validate our model. Due to their strong anticancer efficacy, most chemotherapeutic drugs showed effective on both the 2D and 3D models. Only 17.6% of the drugs with weak or moderate potency exhibited significant difference in efficacy between the 2D and 3D model. In the 3D micro-environment, the heterogeneity of the tumor cells may be a mechanism for drug resistance to the chemotherapeutic drugs (Vincan et al., 2007; Yao et al., 2011). Additionally, the small molecule targeted drugs showed expected sensitivity and higher specificity on the 3D model compared with the corresponding 2D model. Interestingly, the EGFR mutant lung cell lines showed more sensitive to the drugs targeting EGFR on the 3D tumor model. The increased sensitivity on the 3D model suggested that the EGFR expression and downstream signaling may be altered under different culture conditions (Howes et al., 2014; Ayuso et al., 2019). In addition, the antibody-drug conjugate drug of the T-DM1 showed increased resistance on the 3D tumor model that indicated its efficacy was mainly associated with the chemotherapeutic mechanism of action. More importantly, the target drugs showed higher specificity on the 3D tumor model that may reduce false-positive results. Overall, the various drug response under different culture conditions indicated the mechanistic changes in the pathways.

Finally, the *in vivo* CDX model as a standard was applied to further validate the predictive results from the 2D and 3D tumor models. We chose the drug dosages commonly used in the literature. Take the docetaxel as an example, 5–15 mg/kg dosage was recommended previously (Liu et al., 2012; Wallin et al., 2012; Tan et al., 2015). In our study, we selected 10 mg/kg dosage in the CDX models, and the Cmax was 3.465 ± 1.448 μg/ml in mice (Shi et al., 2015). 5 μM docetaxel was used for the *in vitro* models, which was close to the Cmax in mice. The inhibition rate of the docetaxel on the 2D cultured Mia-Paca2 (Pancreatic cancer cell line) model was 65.84%, whereas that on the 3D cultured Mia-Paca2 model was 42.52%. Thus, 5 μM docetaxel was effective on the 2D model, however, exhibited ineffective on the 3D model. The docetaxel-treated Mia-Paca2 xenograft models also showed ineffective with 60.2% of T/C. Nevertheless, the docetaxel-treated H1437, H1838, H1975, H2228 (Non-small cell lung cancer cell lines) xenograft models exhibited effective at <10 mg/kg dosage (Liu et al., 2012; Proia et al., 2012). In addition, non-small cell lung cancer, but not pancreatic cancer, is the indication of the docetaxel in clinical practice. Therefore, 10 mg/kg dosage of the docetaxel was not low for the CDX models, and its negative effect seen *in vivo* was not due to its low dosage. More importantly, the results demonstrated that the drug responses from the 3D tumor model on the array chip were more consistent with the *in vivo* CDX model, and 95% of the false-positive results from the 2D model was excluded. Therefore, we anticipate this matrigel-based 3D micro-tumor model on the array chip can reduce the overuse of animal model, and accelerate anticancer drug development.

It is noted that the commonly used matrigel on our 3D tumor model still has some drawbacks, such as non-quantified impurities and batch-to-batch variations in the mechanical and biochemical properties (Vukicevic et al., 1992; Benton et al., 2011; Aisenbrey and Murphy, 2020). Development of synthetic scaffolds is an emerging direction to provide a controllable and reproducible microenvironment for the tumor cells. Our 3D model recapitulated *in vivo* tumor microenvironment, including tumor cell and tumor cell interaction, as well as tumor cell and extracellular matrix interaction. However, the interaction between the tumor cell and other cell types was not involved so far. The promising future is the establishment of complex co-culture models (e.g., the addition of immune cells or cancer-associated fibroblasts). Patient-derived tumor organoid (PDTO) model is also a superior 3D model because it provides a more physiologically relevant tumor microenvironment. The PDTO is derived from stem cells or progenitor cells, exhibiting cell heterogeneity in gene expression and cellular phenotype. Our 3D tumor model consisting 3D cell aggregates generates from a single cell type. Moreover, the internal development processes drive the PDTO formation, and the 3D tumor model generates *via* cell-to-cell adhesion. However, the 3D tumor model is cost effective compared with the PDTO model. The 2D model also has the advantages in low cost and easy manipulation. Model choice may vary depending on the specific contexts of use. We believe the automated and miniaturized system are the most promising road to overcome the disadvantages of the 3D model.

## Conclusion

In conclusion, we have developed a robust 3D tumor model on an array chip that presents higher accuracy in predicting drug efficacy of the animal model compare to the 2D model by evaluating the chemotherapeutic and targeted drugs. In the large-scale drug evaluation, the chemotherapeutic evaluation of 18 drugs against 27 cancer cell lines showed higher drug resistance on the 3D tumor model. Moreover, the evaluation results of 20 targeted drugs against 17 cancer cell lines showed expected sensitivity and higher specificity on the 3D tumor model compared with the corresponding 2D model. Finally, the evaluation results on the 3D tumor model were more consistent with the *in vivo* cell-derived xenograft model, and excluded 95% false-positive results from the 2D model. We anticipate our 3D tumor model on the array chip being a promising tool for anticancer drug evaluation with widespread acceptance in the drug industry.

## Data Availability

The raw data supporting the conclusions of this article will be made available by the authors, without undue reservation.
