# Supplementary material for: Establishment and large-scale validation of a three-dimensional tumor model on an array chip for anticancer drug evaluation
Source: Front Pharmacol. 2022 Oct 12;13:1032975. doi: 10.3389/fphar.2022.1032975 (PMC9596801; doi:10.3389/fphar.2022.1032975)
Supplement: Supplementary file 1 [file DataSheet1.docx]

Establishment and large-scale validation of a three-dimensional tumor model on an array chip for anticancer drug evaluation

Rong-Rong Xiao^1^, Lei Jin^3^, Nan Xie^3^, Piaopiao Luo^1^, Wenjie Gao^3^, Pengfei Tu^2^, Xiaoni Ai^2*^

^1^ R&D Department, Beijing Daxiang Biotech Co., Ltd., Beijing, China

^2^ State Key Laboratory of Natural and Biomimetic Drugs, School of Pharmaceutical Sciences, Peking University, Beijing, China

^3^ Oncology and Immunology Unit, WuXi Biology, WuXi AppTec (Shanghai) Co., Ltd., Shanghai, China

*** Correspondence:**Xiaoni Ai
[aixn@bjmu.edu.cn](mailto:aixn@bjmu.edu.cn) (X.A.);

Supplementary Material

**
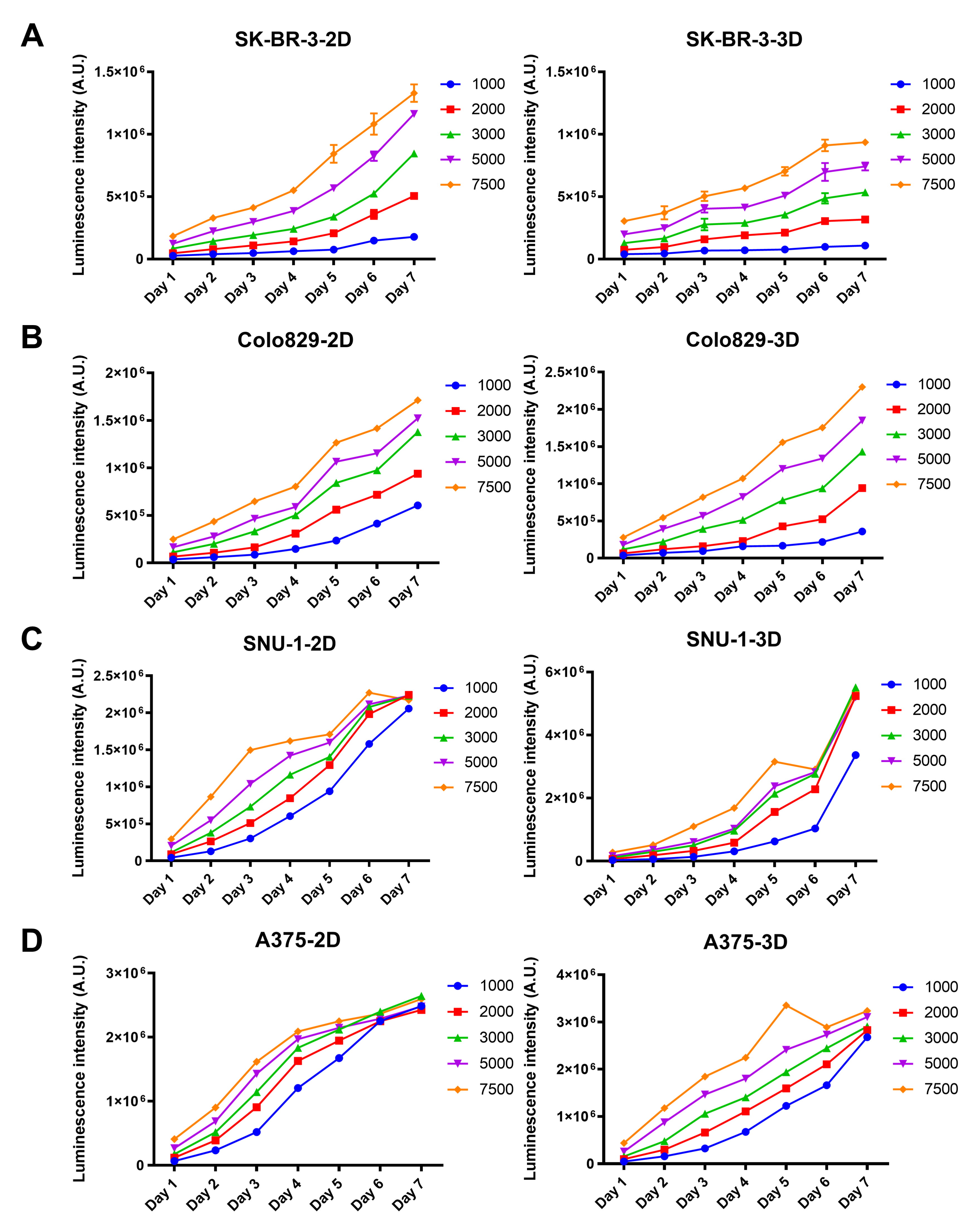
**

**Supplementary Figure 1.** Cell viability of SK-BR-3, HCC116, SNU-1 and A375, which was indicated by luminescence intensity, was measured using CellTitier-Glo from day 1 to 7 in the 2D and 3D models at different seeding densities.


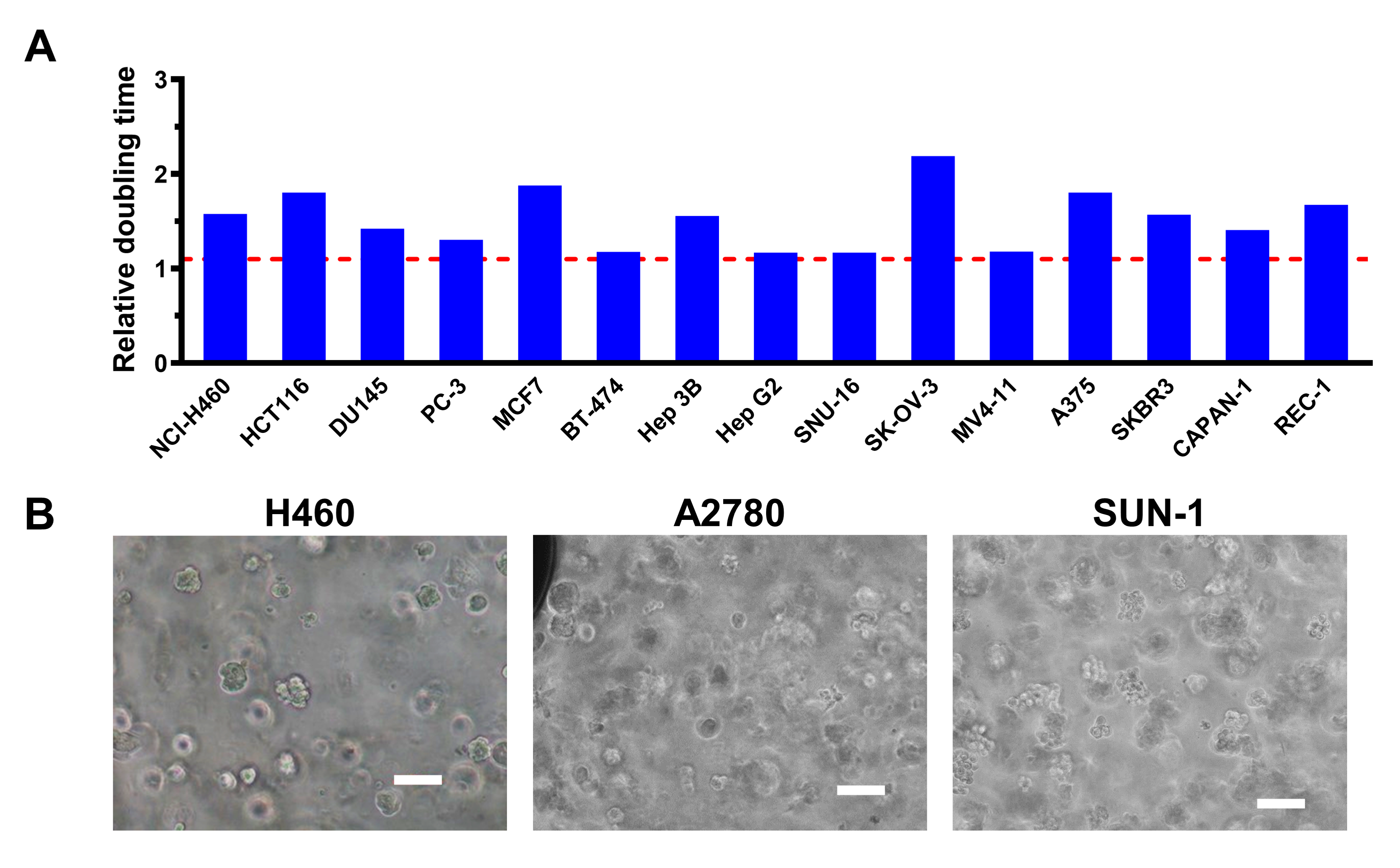


**Supplementary Figure 2.** (A) The relative doubling time of cells in the 3D model compared to the 2D model. (B) The bright field images of NCI-H460, A2780 and SUN-1 on the 3D model after culturing for 4 days. Scale bar = 100 µm.


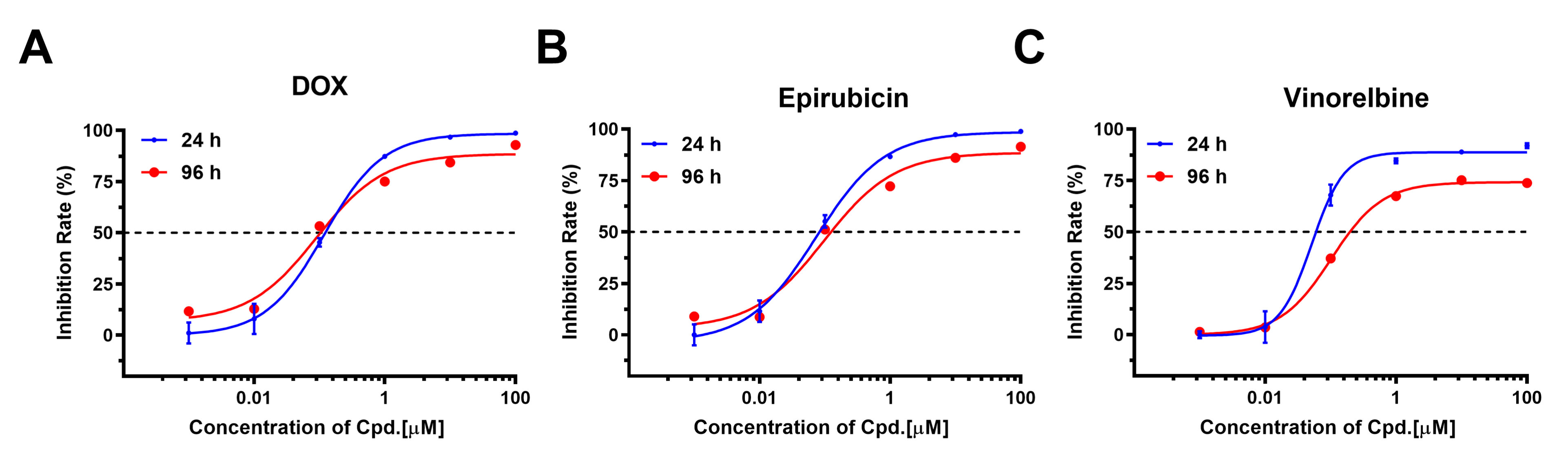


**Supplementary Figure 3.** The dose-effect curves of different chemotherapeutic drugs exert on the 3D model with different pre-culture periods. (A) The dose-effect curves of DOX-treated cell models with the pre-culture time of 24h and 96h. (B) The dose-effect curves of Epirubicin-treated cell models with the pre-culture time of 24h and 96h. (C) The dose-effect curves of Vinorelbine-treated cell models with the pre-culture time of 24h and 96h.


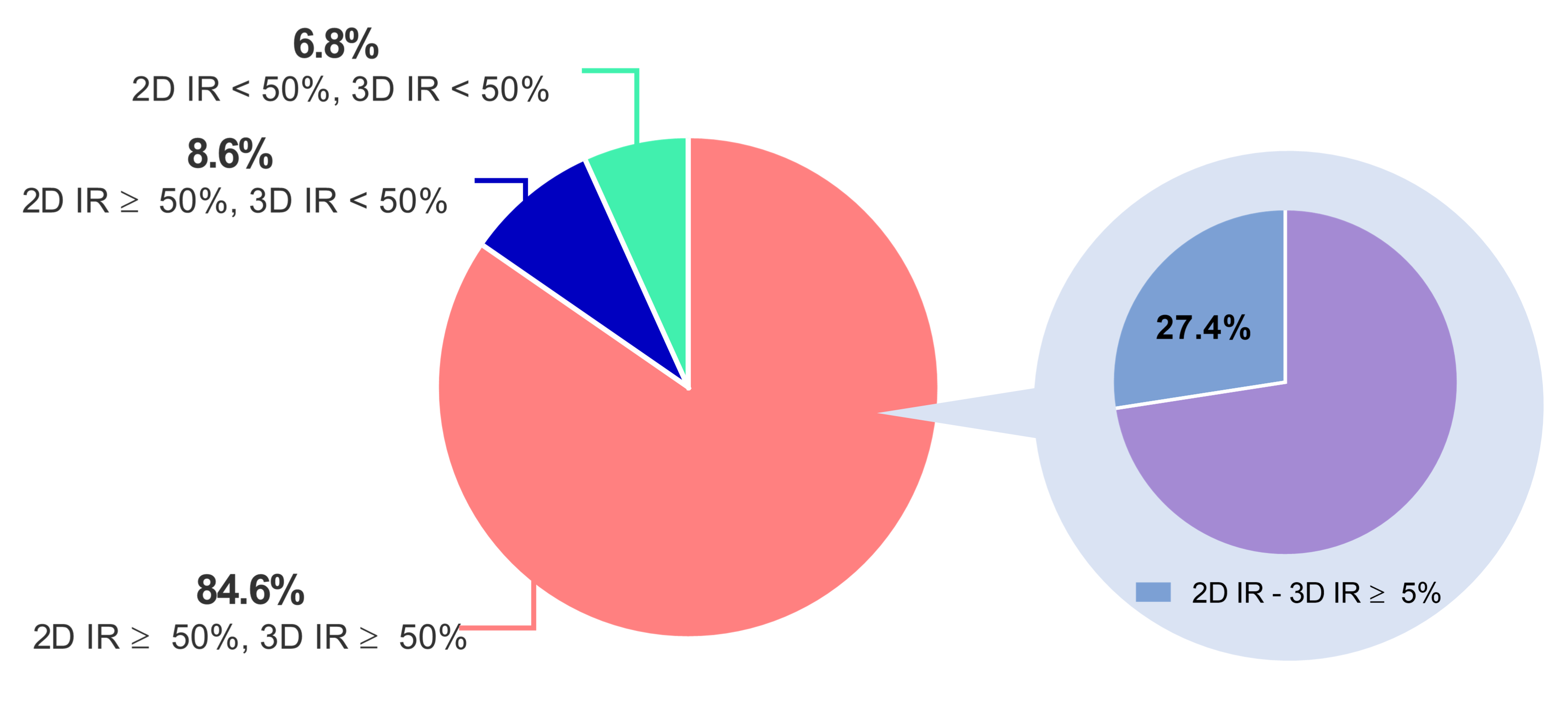


**Supplementary Figure 4.** Primary evaluation results on the 3D and 2D models treated by drugs at the working concentrations showing in Table S3. IR represents inhibition rate.

. **
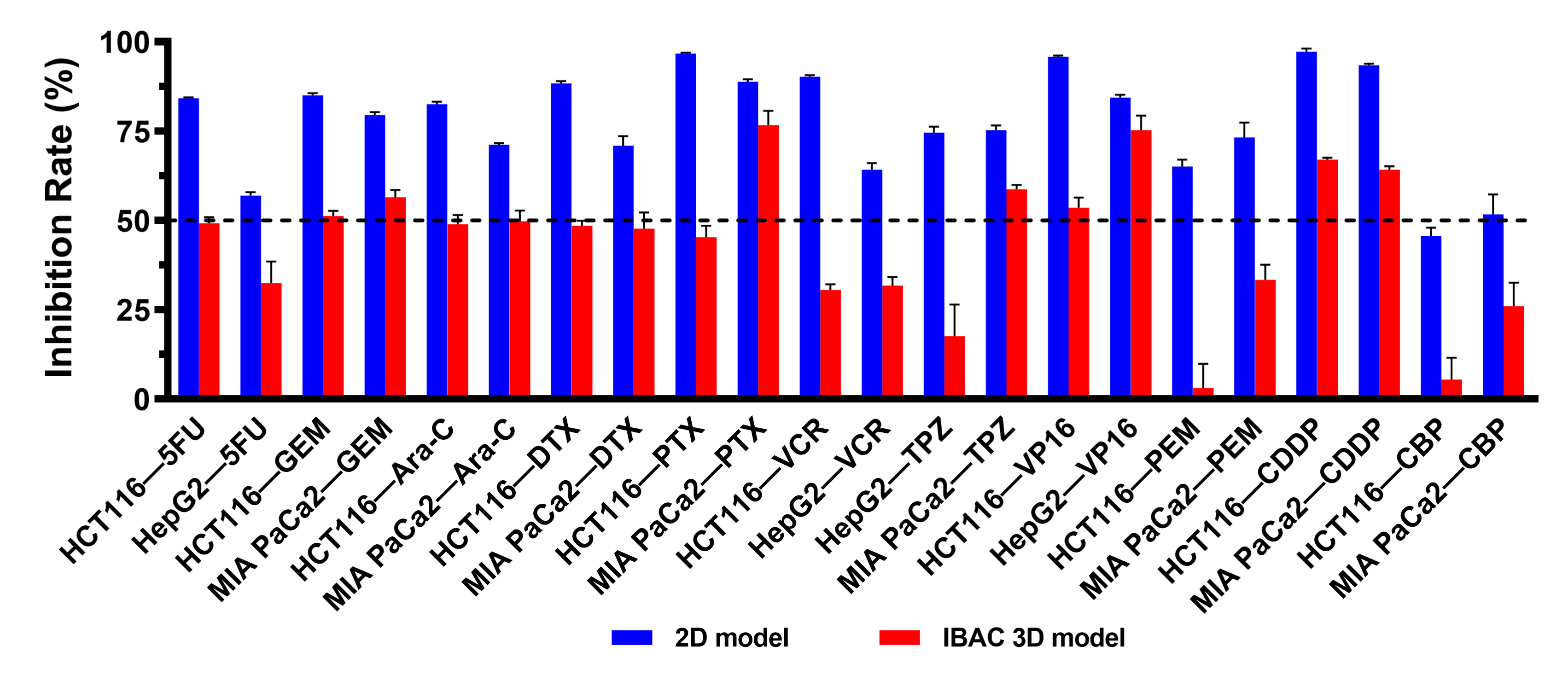
**

**Supplementary Figure 5.** The representative results of primary chemotherapy evaluation to show the inhibition rates of 5-FU, GEM, Ara-C, DTX, PTX, VCR, TPZ, VP16, PEM, CDDP and CBP in colon cancer cell line HCT116, liver cancer cell line HepG2 and pancreatic cancer cell line MIA-PACA2 at single dose. The blue bars showed the inhibition rates on the 2D model, while the red bars showed the inhibition rates on the 3D model. The working concentrations of the chemotherapeutic drugs showing in Table S3.


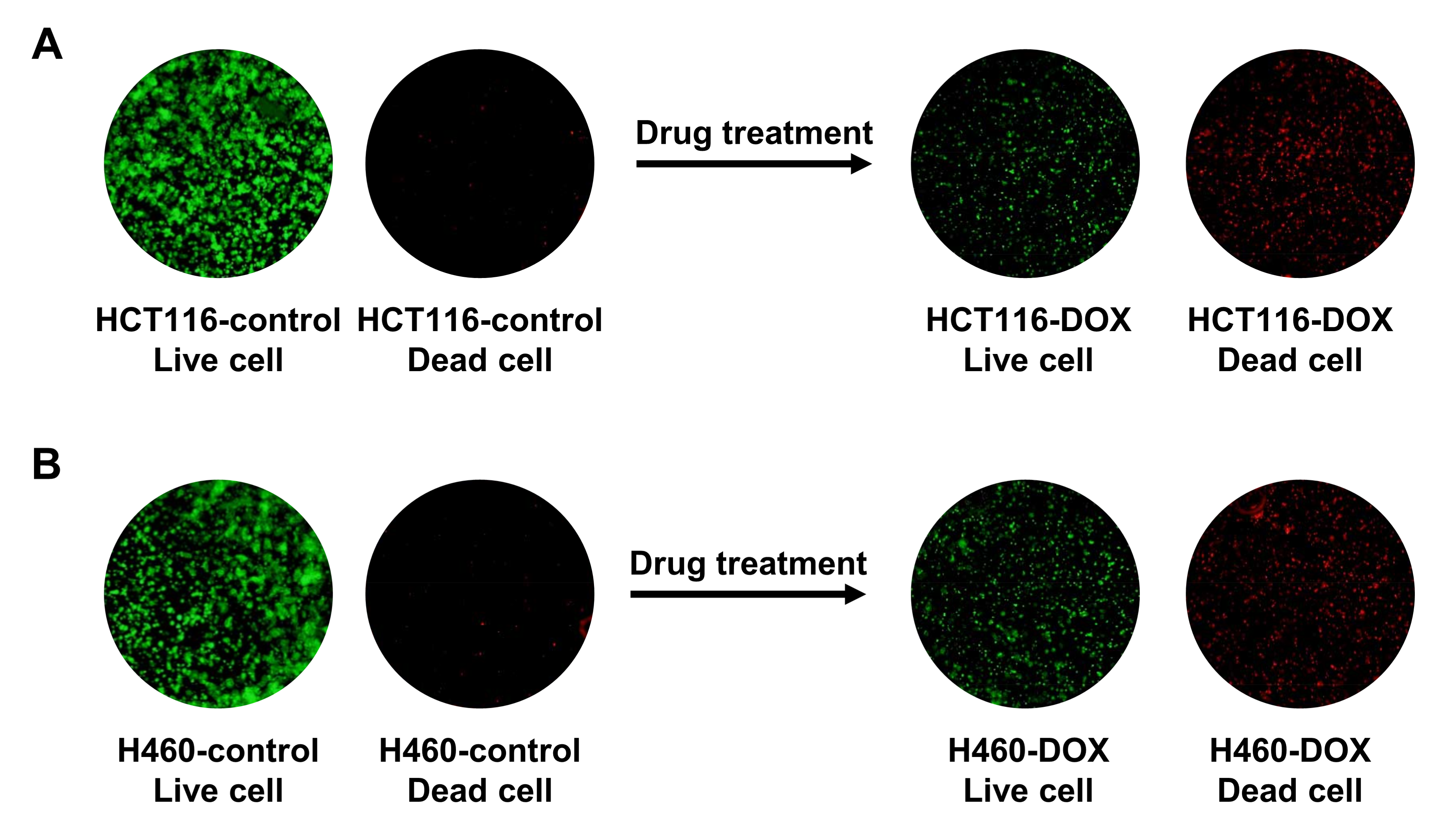


**Supplementary Figure 6.** The drug efficacy was evaluated by staining live cells and dead cells. (A) The results of live/dead staining before and after the DOX treatment on 3D-HCT116 model. (B) The results of live/dead staining before and after the DOX treatment on the 3D-H460 model. Live cells were stained green and dead cells were stained red.

**
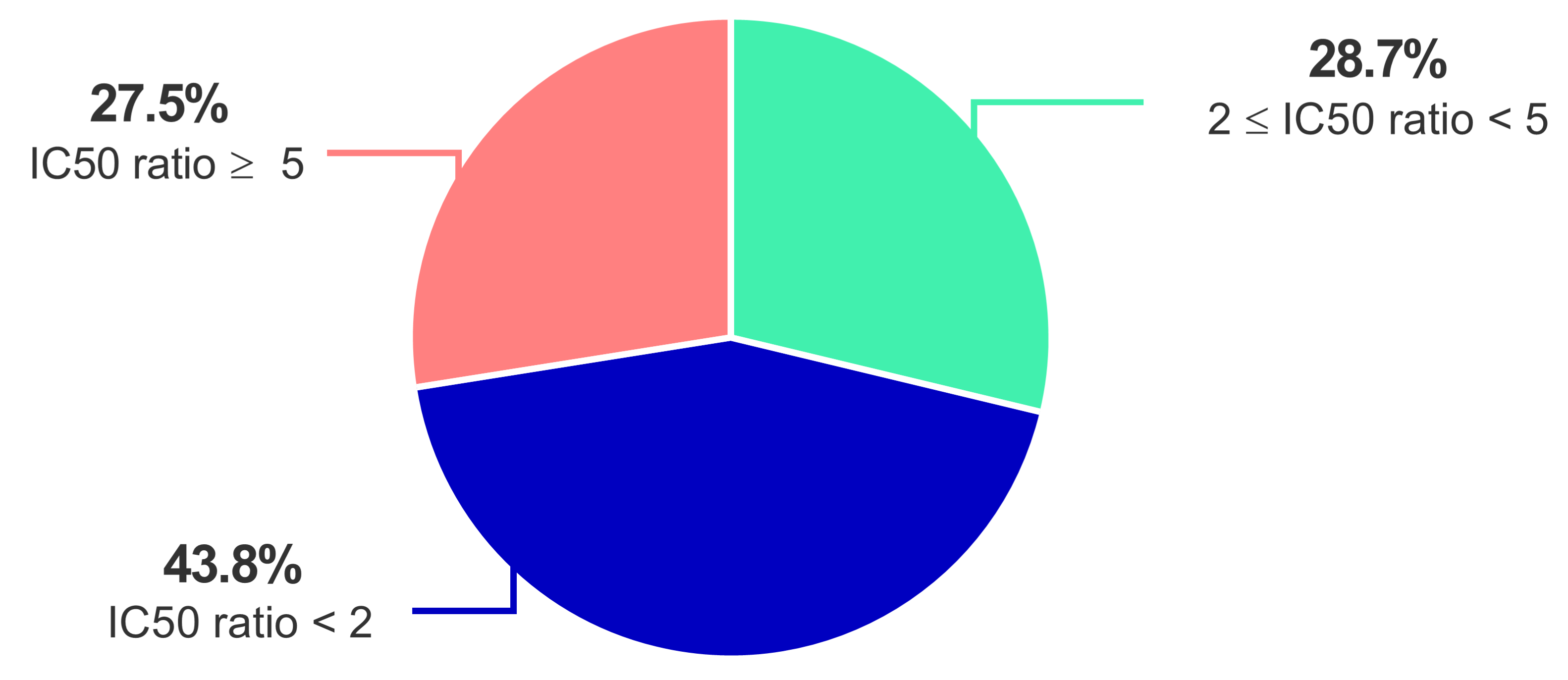
**

**Supplementary Figure 7.** The statistical analysis of secondary drug sensitivity evaluation results after the 3D and 2D models were treated by drugs at serial dilutions. The IC50 values were calculated according to the dose-effect curves.


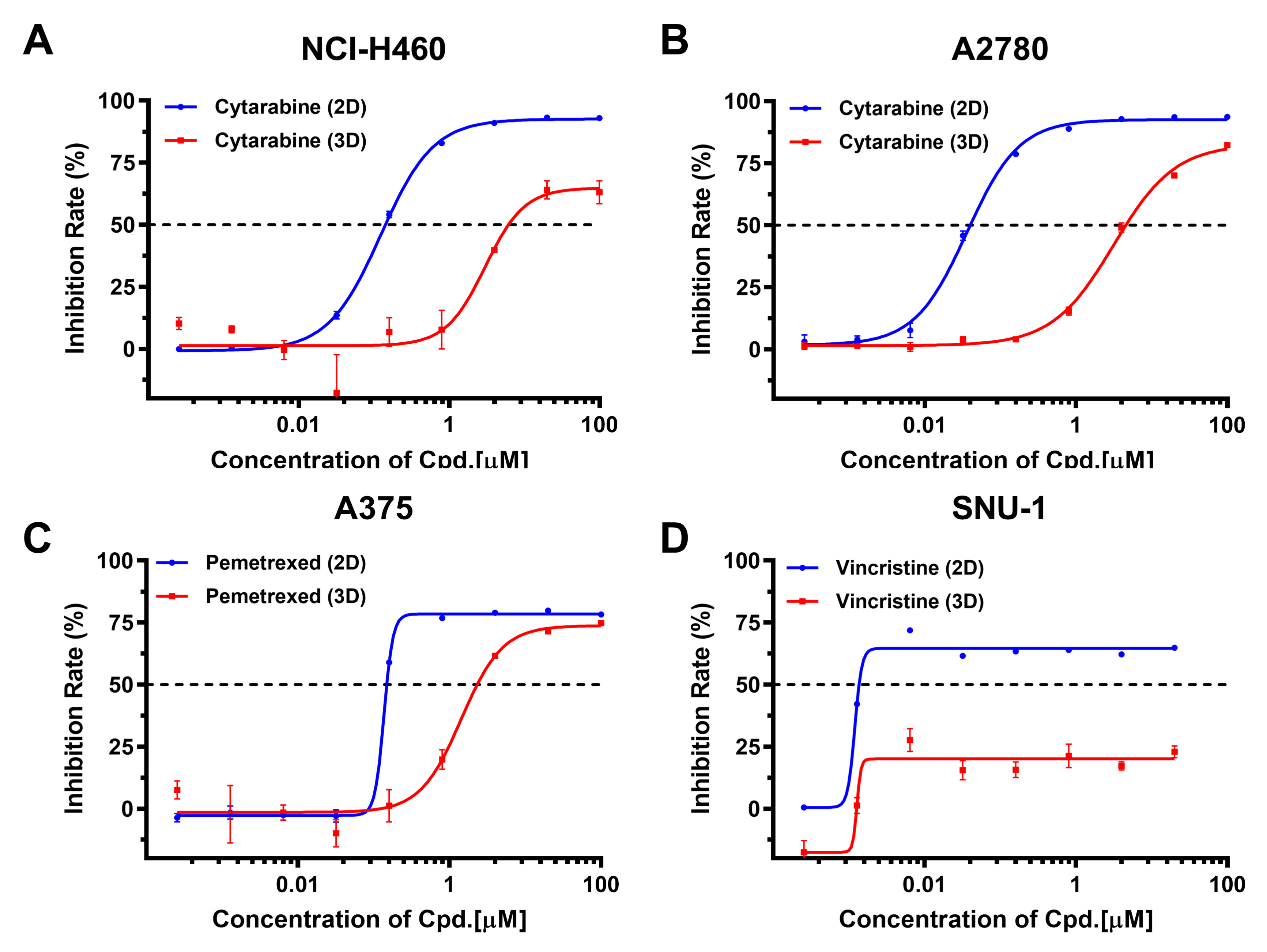


**Supplementary Figure 8.** The comparison of IC50 curves of secondary evaluation of representative chemotherapy responses on the 3D model and the 2D model. (A) The dose-effect curves of NCI-H460 under Cytarabine treatment on the secondary evaluation. (B) The dose-effect curves of A2780 under Cytarabine treatment on the secondary evaluation. (C) The dose-effect curves of A375 under Pemetrexed treatment on the secondary evaluation. (D) The dose-effect curves of SNU-1 under Vincristine treatment on the secondary evaluation.


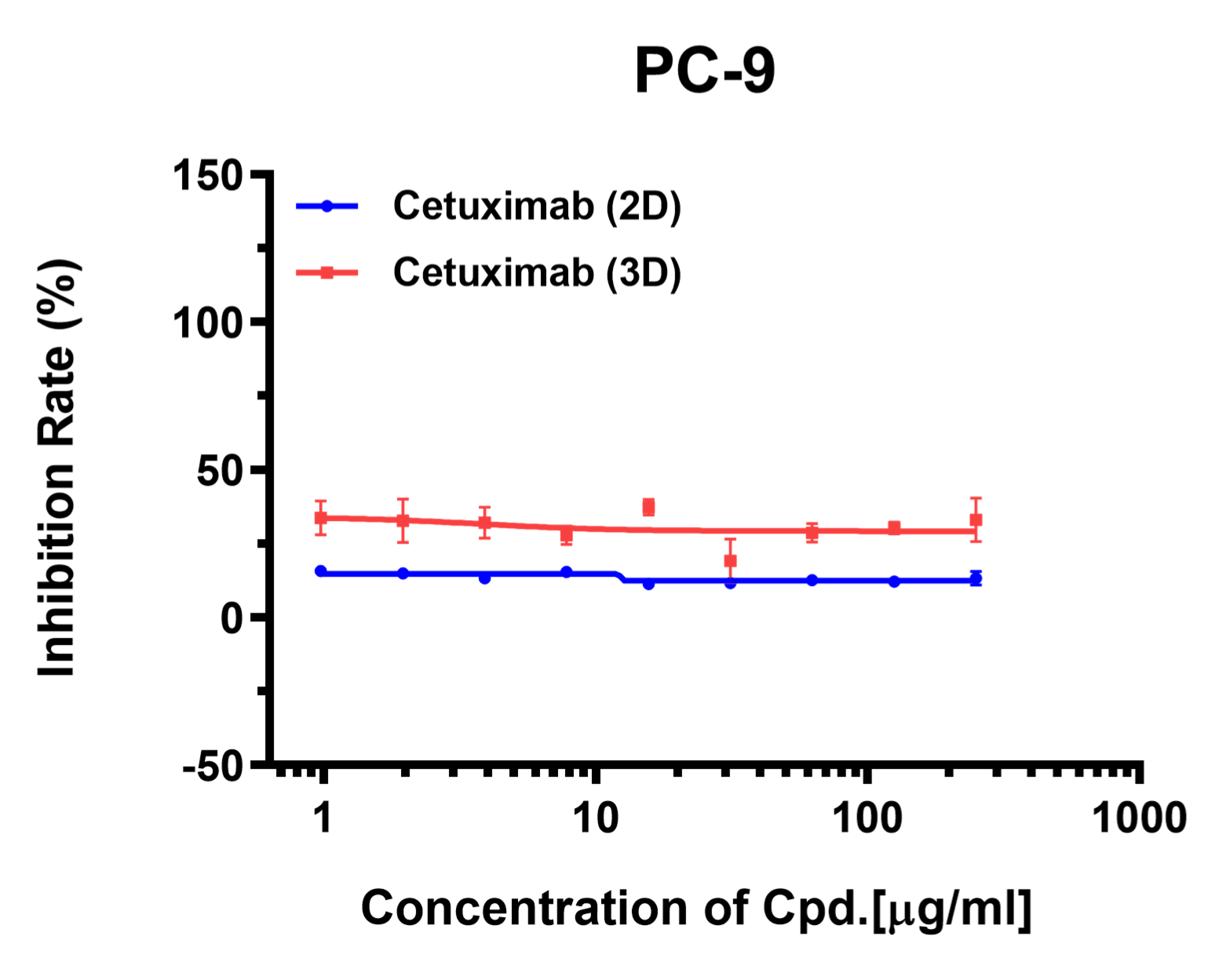


**Supplementary Figure 9.** The dose-effect curves of 2D-PC-9 and 3D-PC9 models after Cetuximab treatment. The cetuximab was tested by 9 concentration points at 2-fold serial dilution, including 250, 125, 62.5, 31.25, 15.62, 7.81, 3.91, 1.95, 0.97 μg/ml.


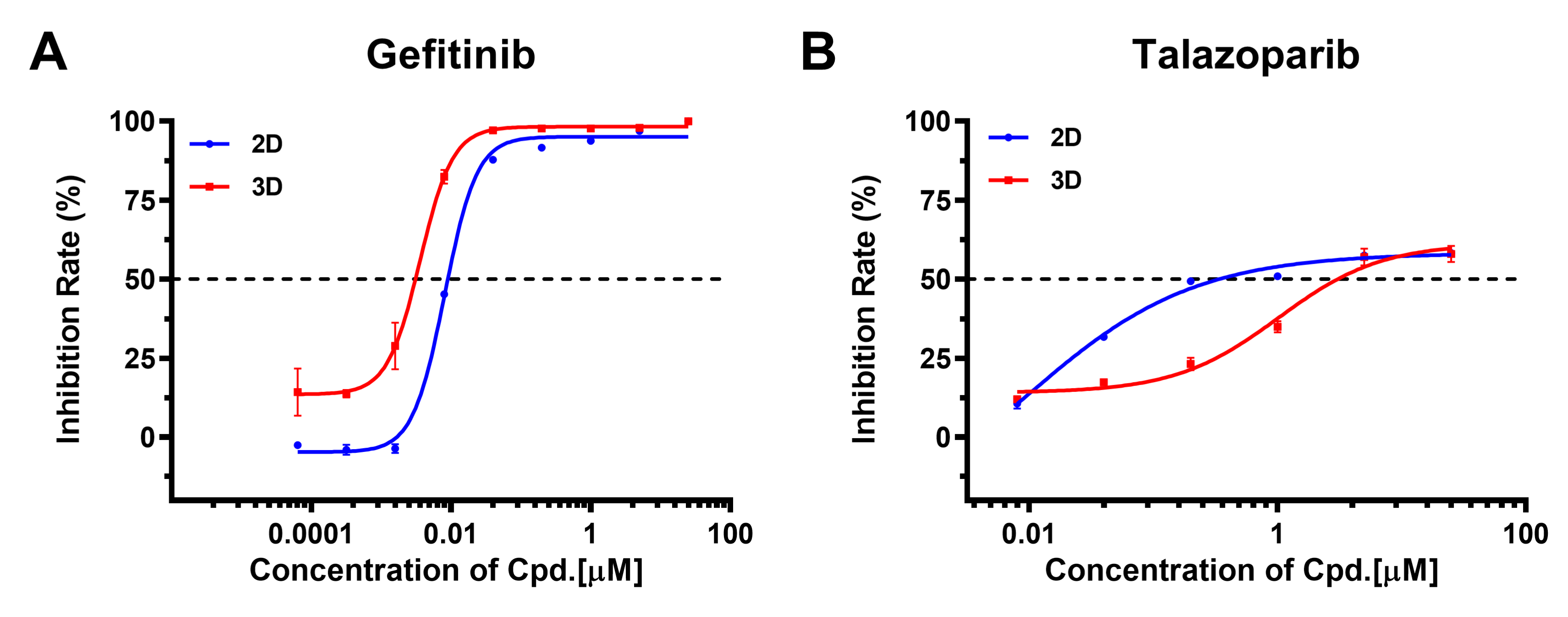


**Supplementary Figure 10.** The representative cross-evaluation comparison of small molecular drugs on the 2D and 3D models. (A) The dose-effect curves of 2D-HCC827 and 3D- HCC827 models after being treated by targeted drug Gefitinib and non-target drug Talazoparib.


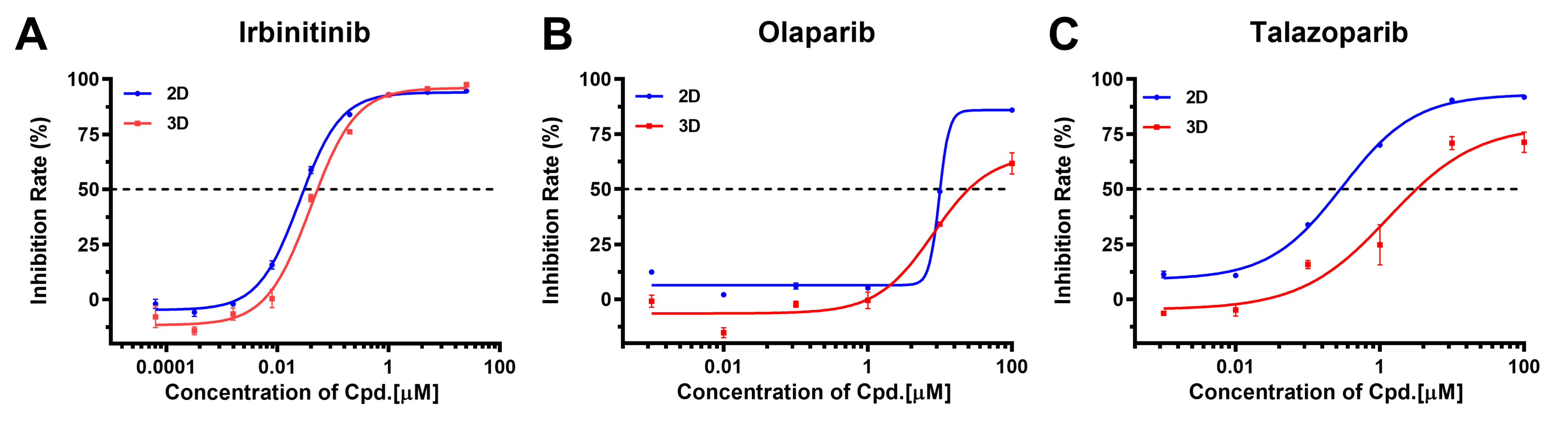


**Supplementary Figure 11.** The representative cross-evaluation comparison of small molecular drugs on the 2D and 3D models. (A) The dose-effect curves of 2D-SKBR3 and 3D-SKBR3 models after being treated by targeted drug Irbinitinib and non-target drug Olaparib and Talazoparib.

**Supplementary Table 1.** Cancer cell lines information.

|  | **Cell Line** | **Cancer Type** |
| --- | --- | --- |
| 1 | NCI-H460 | Lung cancer |
| 2 | NCI-H226 | Lung cancer |
| 3 | HCC827 | Lung cancer |
| 4 | HCT116 | Colorectal cancer |
| 5 | SW620 | Colorectal cancer |
| 6 | HCT15 | Colorectal cancer |
| 7 | DU145 | Prostate cancer |
| 8 | PC-3 | Prostate cancer |
| 9 | MDA-MB-231 | Breast cancer |
| 10 | MCF7 | Breast cancer |
| 11 | BT-474 | Breast cancer |
| 12 | BxPC-3 | Pancreatic cancer |
| 13 | Mia-Paca2 | Pancreatic cancer |
| 14 | Hep 3B | Liver cancer |
| 15 | BEL7404 | Liver cancer |
| 16 | Huh7 | Liver cancer |
| 17 | Hep G2 | Liver cancer |
| 18 | SNU-16 | Gastric cancer |
| 19 | SNU-1 | Gastric cancer |
| 20 | SK-OV-3 | Ovarian cancer |
| 21 | A2780 | Ovarian cancer |
| 22 | Farage | Lymphoma |
| 23 | MV4-11 | Leukemia |
| 24 | MM.1S | Multiple myeloma |
| 25 | RPMI-8226 | Myeloma |
| 26 | A375 | Melanoma |
| 27 | COLO 829 | Melanoma |
| 28 | NCI-H1975 | Lung |
| 29 | PC-9 | Lung |
| 30 | HCC1954 | Breast cancer |
| 31 | AU565 | Breast cancer |
| 32 | SKBR3 | Breast cancer |
| 33 | HCC78 | Lung |
| 34 | MDA-MB-436 | Breast cancer |
| 35 | CAPAN-1 | Pancreatic |
| 36 | REC-1 | Lymphoma |
| 37 | HEL92.1.7 | Hematopoietic |
| 38 | KM-12 | Colorectal cancer |
| 39 | HT29 | Colorectal cancer |

**Supplementary Table 2.** Cancer cell lines and the panel of drugs in targeted drug evaluation.

|  | **Cell Line** | **Species** | **Cancer Type** | **Mutation gene** | **Target Drugs** |
| --- | --- | --- | --- | --- | --- |
| 1 | BT-474 | human | Breast cancer | HER2 | Irbinitinib Lapatinib Trastuzumab T-DM1 |
| 2 | HCC1954 | human | Breast cancer |  |  |
| 3 | AU565 | human | Breast cancer |  |  |
| 4 | SKBR3 | human | Breast cancer |  |  |
| 5 | HCC827 | human | Lung caner | EGFR | Gefitinib Erlotinib Afatinib AZD9291 Cetiximab |
| 6 | NCI-H1975 | human | Lung cancer |  |  |
| 7 | PC-9 | human | Lung cancer |  |  |
| 8 | Huh7 | human | Liver cancer | VEGF | Sunitinib Bevacizumab |
| 9 | HCT15 | human | Colorectal cancer |  |  |
| 10 | MDA-MB-436 | human | Breast cancer | PARP | Olaparib Talazoparib |
| 11 | CAPAN-1 | human | Pancreatic cancer |  |  |
| 12 | KM-12 | human | Colorectal cancer | TRK | LOXO-101 |
| 13 | Farage | human | Lymphoma | PI3Kδ | Idelalisib |
| 14 | HEL92.1.7 | human | Erythroleukemia | JAK1/2 | Ruxolitinib |
| 15 | MCF7 | human | Breast cancer | CDK4/6 | Palbociclib |
| 16 | HT29 | human | Colorectal cancer | BRAF | Dabrafenib |
| 17 | HCC78 | human | Lung cancer | ALK | Crizotinib Ceritinib |

**Supplementary Table 3.** The information of chemotherapeutic drugs.

|  | **Compound** | **Vendor** | **Dilution factor** | **Solvent** | **Compound stock concentration** | **Compound working concentration** |
| --- | --- | --- | --- | --- | --- | --- |
| 1 | 5-FU | selleck | 5 | DMSO | 40 mM | 100 μM |
| 2 | Gemcitabine | selleck | 5 | DMSO | 40 mM | 100 μM |
| 3 | Mitomycin | selleck | 5 | DMSO | 40 mM | 100 μM |
| 4 | Cytarabine | selleck | 5 | DMSO | 40 mM | 100 μM |
| 5 | Docetaxel | selleck | 5 | DMSO | 10 mM | 25 μM |
| 6 | Paclitaxel | selleck | 5 | DMSO | 4 mM | 10 μM |
| 7 | Vincristine | selleck | 5 | DMSO | 40 mM | 100 μM |
| 8 | Vinorelbine | selleck | 5 | DMSO | 40 mM | 100 μM |
| 9 | Tirapazamine | sigma | 5 | DMSO | 40 mM | 100 μM |
| 10 | Etoposide | selleck | 5 | DMSO | 40 mM | 100 μM |
| 11 | Doxorubicin | selleck | 5 | DMSO | 40 mM | 100 μM |
| 12 | Vorinostat | selleck | 5 | DMSO | 40 mM | 100 μM |
| 13 | Romidepsin | selleck | 5 | DMSO | 40 mM | 100 μM |
| 14 | Bortezomib | selleck | 5 | DMSO | 40 mM | 100 μM |
| 15 | Lxazomib | selleck | 5 | DMSO | 40 mM | 100 μM |
| 16 | Pemetrexed | selleck | 5 | ddH_2_O | 40 mM | 100 μM |
| 17 | Cisplatin | selleck | 5 | Saline | 3 mM | 30 μM |
| 18 | Carboplatin | selleck | 5 | ddH_2_O | 6 mM | 60 μM |
| 19 | Idarubicin | selleck | 5 | DMSO | 40 mM | 100 μM |

**Supplementary Table 4.** The information of target drugs.

| **Target** | **Compound** | **Vendor** | **Dilution factor** | **Solvent** | **Compound stock concentration** | **Compound working concentration** |
| --- | --- | --- | --- | --- | --- | --- |
| HER2 | Irbinitinib | selleck | 5 | DMSO | 10000 μM | 25 μM |
|  | Lapatinib | selleck | 5 | DMSO | 10000 μM | 25 μM |
|  | Trastuzumab | selleck | 2 | PBS | 5000 μg/mL | 250 μg/mL |
|  | T-DM1 | Genentech | 5 | PBS | 20000 μg/mL | 400 μg/mL |
|  | Pertuzuma | selleck | 5 | PBS | 5000 μg/mL | 100 μg/mL |
| EGFR | Gefitinib | selleck | 5 | DMSO | 10000 μM | 25 μM |
|  | Erlotinib | selleck | 5 | DMSO | 10000 μM | 25 μM |
|  | Afatinib | selleck | 5 | DMSO | 10000 μM | 25 μM |
|  | AZD9291 | selleck | 5 | DMSO | 10000 μM | 25μM |
|  | Cetuximab | selleck | 2 | PBS | 5000 μg/mL | 250 μg/mL |
| VEGF | Sunitinib | selleck | / | DMSO | 10000 μM | 25 μM |
|  | Bevacizumab | selleck | / | PBS | 5000 μg/mL | 100 μg/mL |
|  | Sorafenib | selleck | / | DMSO | 10000 μM | 25 μM |
| PARP | Olaparib | selleck | 5 | DMSO | 40000 μM | 100 μM |
|  | Talazoparib | selleck | 5 | DMSO | 10000 μM | 25 μM |
| TRK | LOXO-101 | selleck | 5 | DMSO | 40000 μM | 100 μM |
| PI3Kδ | Idelalisib | selleck | 5 | DMSO | 40000 μM | 100 μM |
| JAK1/2 | Ruxolitinib | selleck | 5 | DMSO | 40000 μM | 100 μM |
| CDK4/6 | Palbociclib | selleck | 5 | H_2_O | 40000 μM | 100 μM |
| BRAF | Dabrafenib | selleck | 5 | DMSO | 40000 μM | 100 μM |
| ALK | Crizotinib | selleck | 5 | DMSO | 40000 μM | 100 μM |
|  | Ceritinib | selleck | 5 | DMSO | 10000 μM | 25 μM |
| BTK | Ibrutinib | selleck | 5 | DMSO | 10000 μM | 25 μM |

**Supplementary Table 5.** The information of drug administration on the CDX model.

|  | **Mouse strains** | **Models** | **Drugs** | **Dosage (mg/kg)** | **Route** | **Schedule** |
| --- | --- | --- | --- | --- | --- | --- |
| 1 | nu/nu | NCI-H460 | 5-FU | 15 | - | QD x 14D |
| 2 | nu/nu | NCI-H460 | Pemetrexed | 150 | i.p. | QD x 5, for two cycles |
| 3 | BALB/c nude | HCC827 | Pemetrexed | 250 | i.p. | QW x 3W |
| 4 | nu/nu | HCT-116 | 5-FU | 70 | i.p. | Q2W |
| 5 | SCID | HCT-116 | Cytarabine | 13.3 | i.p. | Q4H x 3, day 6–14 |
| 6 | nu/nu | HCT-116 | Docetaxel | 5 | i.p. | Q2W |
| 7 | BALB/c nude | HCT-116 | Paclitaxel | 10 | i.v. | - |
| 8 | BALB/c nude | HCT-116 | Vincristine | 1.6 | i.v. | QD x 1 |
| 9 | BALB/c nude | HCT-116 | Pemetrexed | 50 | i.p. | (QDx5) x 21D |
| 10 | NCr-nu athymic mice | SW620 | Cytarabine | 25 | i.p. | Q4H x 6, days 9,13,17 |
| 11 | BALB/c nude | SW620 | Vincristine | 0.5 | i.v. | QW x 3W |
| 12 | BALB/c nude | SW620 | Tirapazamine | 25 | i.p. | Q2D x 21D |
| 13 | BALB/c nude | HCT15 | 5-FU | 50 | i.v. | Q4D x 3 |
| 14 | BALB/c nude | HCT15 | Gemcitabine | 40 | i.v. | QW x 2 |
| 15 | BALB/c nude | HCT15 | Pemetrexed | 50 | i.p. | (QDx5) x 21D |
| 16 | Athymic nude | PC-3 | 5-FU | 60 | i.p. | QD x 4D |
| 17 | SCID | MDA-MB-231 | Paclitaxel | 10 | i.p. | on days 5 and 10 |
| 18 | BALB/cj | MDA-MB-231 | Tirapazamine | 25 | i.p. | QW |
| 19 | BALB/c nude | MCF7 | Pemetrexed | 20 | i.v. | QD x 10D |
| 20 | BALB/c nude | BxPC-3 | Pemetrexed | 50 | i.p. | (QDx5) x 21D |
| 21 | CD-1 nu/nu | Mia-Paca2 | Cytarabine | 40 | i.p. | QD x 10D |
| 22 | BALB/c nude | Mia-Paca2 | Docetaxel | 10 | i.p. | QW x 3W |
| 23 | BALB/c nude | Mia-Paca2 | Pemetrexed | 50 | i.p. | (QDx5) x 21D |
| 24 | BALB/c nude | Hep3B | Pemetrexed | 50 | i.p. | (QDx5) x 21D |
| 25 | BALB/c nude | BEL7404 | Cytarabine | 50 | i.p. | (QDx5) x 21D |
| 26 | BALB/c nude | BEL7404 | Tirapazamine | 25 | i.p. | Q2D x 21D |
| 27 | Athymic nude | Hep G2 | 5-FU | 40 | i.p. | QD |
| 28 | BALB/c nude | Hep G2 | Vincristine | 0.4 | i.p. | day 5 |
| 29 | Athymic nude | Hep G2 | Tirapazamine | 30 | i.p. | Q2D |
| 30 | BALB/c nude | SNU-1 | Paclitaxel | 20 | i.p. | QW x 3W |
| 31 | BALB/c nude | SNU-1 | 5FU | 30 | i.p. | TIW x 4W |
| 32 | BALB/c nude | SNU-1 | Docetaxel | 10 | i.p. | QW x 3W |
| 33 | BALB/c nude | SNU-1 | Pemetrexed | 50 | i.p. | (QDx5) x 21D |
| 34 | BALB/c nude | SNU-1 | Carboplatin | 75 | i.p. | QW x 4W |
| 35 | BALB/c nude | SK-OV-3 | 5-FU | 50 | i.v. | Q4D x 3 |
| 36 | nu/nu | A2780 | Carboplatin | 60 | i.p. | day 0 |
| 37 | BALB/c nude | A2780 | Pemetrexed | 50 | i.p. | (QDx5) x 14D |
| 38 | CB-17 SCID | MM.1S | Pemetrexed | 50 | i.p. | (QDx5) x 21D |
| 39 | CB-17 SCID | RPMI-8226 | Pemetrexed | 50 | i.p. | (QDx5) x 21D |
| 40 | BALB/c nude | A375 | Tirapazamine | 25 | i.p. | Q2D x 21D |

**Supplementary Table 6.** The IC50 values and inhibition rates of the EGFR-mutant cell lines after administrated with the EGFR-targeting drugs.

|  | **Compound** | **Efficacy** | **H1975**  **2D** | **H1975**  **3D** | **PC-9**  **2D** | **PC-9**  **3D** | **HCC827**  **2D** | **HCC827**  **3D** |
| --- | --- | --- | --- | --- | --- | --- | --- | --- |
| 1 | Gefitinib | IC_50_ (μM)  IR | 9.538  87.2% | 12.400  77.3% | 0.016  100% | 0.010  99.9% | 0.009  100% | 0.003  99.9% |
| 2 | Erlotinib | IC_50_ (μM)  IR | 8.533  68.0% | 8.224  76.6% | 0.016  98% | 0.013  99.8% | 0.016  98.2% | 0.013  98.5% |
| 3 | Afatinib | IC_50_ (μM)  IR | 0.170  99.99% | 0.108  99.8% | 0.001  90.13% | 0.001  98.06% | 0.002  100% | 0.001  100% |
| 4 | AZD9291 | IC_50_ (μM)  IR | 0.018  100% | 0.013  100% | 0.006  100% | 0.005  100% | 0.009  100% | 0.003  100% |
| 5 | Cetuximab | IC_50_(μg/ml)  IR | >250  6.20% | >250  21.65% | >250  16.15% | >250  26.11% | >250  39.8% | <0.97  74.7% |

Note: IR represents inhibition rate from the primary evaluation. High IC_50_ value and low IR indicate poor efficacy, whereas low IC_50_ value and high IR indicate strong potency.
